# A genome sequence for *Biomphalaria pfeifferi*, the major vector snail for the human-infecting parasite *Schistosoma mansoni*

**DOI:** 10.1371/journal.pntd.0011208

**Published:** 2023-03-24

**Authors:** Lijing Bu, Lijun Lu, Martina R. Laidemitt, Si-Ming Zhang, Martin Mutuku, Gerald Mkoji, Michelle Steinauer, Eric S. Loker

**Affiliations:** 1 Department of Biology, Center for Evolutionary and Theoretical Immunology, Parasite Division Museum of Southwestern Biology, University of New Mexico, Albuquerque, New Mexico, United States of America; 2 Center for Biotechnology Research and Development, Kenya Medical Research Institute, Nairobi, Kenya; 3 College of Osteopathic Medicine of the Pacific–Northwest, Western University of Health Sciences, Lebanon, Oregon, United States of America; NIAID: National Institute of Allergy and Infectious Diseases, UNITED STATES

## Abstract

**Background:**

*Biomphalaria pfeifferi* is the world’s most widely distributed and commonly implicated vector snail species for the causative agent of human intestinal schistosomiasis, *Schistosoma mansoni*. In efforts to control *S*. *mansoni* transmission, chemotherapy alone has proven insufficient. New approaches to snail control offer a way forward, and possible genetic manipulations of snail vectors will require new tools. Towards this end, we here offer a diverse set of genomic resources for the important African schistosome vector, *B*. *pfeifferi*.

**Methodology/Principal findings:**

Based largely on PacBio High-Fidelity long reads, we report a genome assembly size of 772 Mb for *B*. *pfeifferi* (Kenya), smaller in size than known genomes of other planorbid schistosome vectors. In a total of 505 scaffolds (N50 = 3.2Mb), 430 were assigned to 18 large linkage groups inferred to represent the 18 known chromosomes, based on whole genome comparisons with *Biomphalaria glabrata*. The annotated *B*. *pfeifferi* genome reveals a divergence time of 3.01 million years with *B*. *glabrata*, a South American species believed to be similar to the progenitors of *B*. *pfeifferi* which undertook a trans-Atlantic colonization < five million years ago.

**Conclusions/Significance:**

The genome for this preferentially self-crossing species is less heterozygous than related species known to be preferential out-crossers; its smaller genome relative to congeners may similarly reflect its preference for selfing. Expansions of gene families with immune relevance are noted, including the *FReD* gene family which is far more similar in its composition to *B*. *glabrata* than to *Bulinus truncatus*, a vector for *Schistosoma haematobium*. Provision of this annotated genome will help better understand the dependencies of trematodes on snails, enable broader comparative insights regarding factors contributing to susceptibility/ resistance of snails to schistosome infections, and provide an invaluable resource with respect to identifying and manipulating snail genes as potential targets for more specific snail control programs.

## Author summary

*Biomphalaria pfeifferi* is the world’s most widely distributed and commonly implicated vector snail for the causative agent of human intestinal schistosomiasis, *Schistosoma mansoni*. Understanding the basis of host-parasite compatibility relies on functional genomic resources, which will be helpful for developing genetic-based control of schistosomes and their vector snails. Here we report a high-quality, *de novo*–assembled whole genome for *B*. *pfeifferi* (PacBio High-Fidelity long reads), with in-depth gene model annotation for protein-coding and nonprotein-coding genes. Using these enriched molecular data, a divergence date of 3.01 Mya was calculated for *B*. *pfeifferi* and *B*. *glabrata*, consistent with the idea of *Biomphalaria* colonization of Africa from South America, and placing an upper boundary on the age of *S*. *mansoni* in Africa. The spread of *S*. *mansoni* was likely favored by its high degree of compatibility with *B*. *pfeifferi*-like snails. In addition, our evidence supports the idea that *B*. *pfeifferi* is a preferentially self-crossing species with a more homozygous and smaller genome than related species like *B*. *glabrata* known to be preferential out-crossers. Furthermore, several immune-related gene families have expanded differently between *B*. *pfeifferi* to *B*. *glabrata*. Resources provided here will deepen our general knowledge of molluscan biology, host-parasite co-evolution, vector biology, and genomics.

## Introduction

*Biomphalaria pfeifferi* (Krauss, 1848) is a freshwater planorbid gastropod widely distributed across sub-Saharan Africa and Madagascar, with populations also found in the Sahara and southwest Asia [1,2]. The impetus to undertake this study comes from the role this snail plays as the major intermediate host species for the human-infecting blood fluke, *Schistosoma mansoni*, the world’s most common causative agent of intestinal schistosomiasis [3].

Schistosomiasis remains entrenched as one of the world’s most prevalent and debilitating neglected tropical diseases (NTDs), infecting over 200 million people, most of whom live in sub-Saharan Africa [4]. At a time when the world’s attention has been understandably focused on emerging viruses, endemic neglected tropical diseases like schistosomiasis have continued to exact their usual, greater-than-appreciated toll on human health [5], aided by the SARS-CoV-2 pandemic which has disrupted or delayed ongoing efforts to treat infected individuals as part of control or elimination efforts [6].

Throughout much of sub-Saharan Africa, the underlying conditions that have always favored the transmission of schistosomiasis remain prevalent. Poverty, associated with poor sanitation, use of surface waters from natural habitats for basic needs, and limited access to health services, continue to enable the transmission of schistosomiasis [7]. Although complex, the life cycle of schistosome parasites is inherently difficult to perturb. Sexually-reproducing and potentially long-lived adult worms exploit ubiquitous and mobile human hosts and diverse species of reservoir hosts. The asexually reproducing schistosome larval stages live in particular and abundant species of freshwater snails like *B*. *pfeifferi*, and release multitudes of free-swimming cercariae into the water that are remarkably effective in finding and penetrating the skin of human hosts. Aquatic habitats and the vector snails living in them lie at the heart of schistosomiasis transmission and as the world’s demands for shrinking aquatic resources increase, there will be profound changes in store for snails and the schistosomes they host [8–10].

Approximately 18 of the world’s 34 species of *Biomphalaria* are known to be susceptible to *S*. *mansoni* infection [11]. In addition to *B*. *pfeifferi*, the related species *B*. *glabrata* is particularly noteworthy, not only because it serves as an important vector of *S*. *mansoni* in the Neotropics, but also because genome resources are available for it [12]. Additionally, *B*. *straminea*, for which a genome sequence has also recently been produced [13], plays a role in hosting *S*. *mansoni* in South America, and has proved to be invasive, having colonized southeast Asia where its role as a potential host for *S*. *mansoni* has become a concern [14,15]. A genome sequence for *Bulinus truncatus*, a related planorbid snail that hosts *Schistosoma haematobium*, the causative agent of urinary schistosomiasis, has also recently been provided [16].

*Biomphalaria pfeifferi* is notable among *Biomphalaria* species for two reasons. Across its broad geographic range, individuals highly susceptible to *S*. *mansoni* infections are present, regardless of the geographic origin of the *S*. *mansoni* isolate being tested [17–20]. This may relate to the peculiar nature of its presumed origins from a trans-Atlantic *Biomphalaria* colonist, and its potential role as an ancestral host for *S*. *mansoni* as the latter arose in Africa [11,21]. Secondly, the population biology of *B*. *pfeifferi* is distinctive because, although capable of out-crossing, it is a strong preferential self-fertilizer [22,23]. This gives rise to natural populations comprised of mixtures of identifiable lineages derived from selfing that in aggregate are limited in genetic diversity relative to populations of outcrossing relatives. Different natural populations are also relatively divergent from one another. Their proclivity for selfing is generally explained as an adaptation for rapidly colonizing fast-changing freshwater environments, such as streams subject to repeated cycles of flooding and drying [23]. The colonization by one or a few lineages of *B*. *pfeifferi* of freshwater habitats on a large area of land reclaimed for agriculture as a result of construction of the Diama dam on the Senegal River contributed to an epidemic of *S*. *mansoni* occurring in the region thereafter [24]. Thus, the rarity of outcrossing in *B*. *pfeifferi* populations may also help explain the high susceptibility of at least some lineages of *B*. *pfeifferi* to potentially fast-evolving parasites like *S*. *mansoni*, but also to several additional trematode species, at least 19 of which are known to infect this snail [25].

Schistosomiasis control efforts are still remarkably unidimensional and rely heavily on treatment of people with praziquantel both to improve individual health and to diminish transmission. The molluscan vectors of schistosomes have long been recognized as a much-needed alternative target for schistosomiasis control efforts, but efforts in this sphere have not proceeded past basic approaches such as the use of molluscicides, habitat modifications or introductions of potential snail predators or competitors [26]. To make any real headway in this endeavor, a more detailed and nuanced understanding of the underlying biology of the vector snails is needed, including of what lies within their genomes, and how the genetic resources of snails like *B*. *pfeifferi* might be exploited to achieve control.

Studies of the transcriptomics responses of *B*. *pfeifferi* exposed to *S*. *mansoni* and molluscicides have been undertaken [27,28], but the genomic resources currently available for *B*. *pfeifferi* are surprisingly limited compared to some other schistosome snail vector species (Table 1). This is particularly noteworthy given the widespread involvement of *B*. *pfeifferi* in transmission of *S*. *mansoni* across its broad regions of endemicity in the Afrotropical region and southwest Asia.

**Table 1:** Current *B*. *pfeifferi* genomic resources are limited as compared to some other relevant schistosome vector species, based on GenBank records, as of August 10, 2022.

Species	Genome Assemblies	Genes	Nucleotides	Proteins	GEO Datasets	PubMed Central	SRA Experiments
***Biomphalaria pfeifferi***	**0**	**35**	**407**	**158**	**0**	**226**	**30**
*Biomphalaria glabrata*	4	30,353	444,181	37,769	285	1,596	1,520
*Biomphalaria straminea*	1	43,340	43,340	40,218	0	160	40
*Bulinus truncatus*	1	26,729	26,729	26,729	0	503	52
*Schistosoma mansoni*	4	19,101	324,653	15,808	1,340	9,204	4,510

**Note:** This table shows the number of records recovered from keyword search queries from multiple National Center for Biotechnology Information (NCBI) databases. The number of genes is not necessarily the same as reported from the corresponding genome papers because other researchers may have deposited additional gene sequences to NCBI before or after the genome resources were submitted to GenBank. GEO, Gene Expression Omnibus, a public repository of microarray and sequence-based gene expression profiles; the PubMed Central column indicates the number of free full-text publications pertaining to *B*. *pfeifferi* in journal literature; SRA, Sequence Read Archive, indicates the publicly available repositories of high throughput sequencing data for *B*. *pfeifferi*.

Our hope is additional genomic information for *B*. *pfeifferi* might be exploited by the scientific community in several ways, to better understand: 1) the nature and consequences of its peculiar population structure, and how it differs from other *Biomphalaria* species in this regard, and how we might manipulate it to our advantage; 2) how *B*. *pfeifferi* responds rapidly to environmental change and how it might respond to looming long-term environmental changes like warming climates or increased pollution; 3) how we might devise specific ways to disrupt snail reproduction or selectively kill schistosome-infected snails; 4) the symbiont community supported by *B*. *pfeifferi*, including potential snail pathogens or essential metabolic partners; 5) how we might alter or enhance the ability of *B*. *pfeifferi* to overcome its susceptibility to schistosome infection, such as by diminishing its attractiveness for schistosome infectious stages or by enhancing immune responsiveness; and 6) add to the growing database of molluscan genomics that will provide increasing general insights into the many unique features of the Phylum Mollusca.

Below we detail the results obtained from application of PacBio HiFi long-read sequencing technology to obtain a high quality and fully annotated genome for *B*. *pfeifferi*, one which has been interrogated for its representation of several gene families believed to be relevant to its role in serving as an intermediate host for *S*. *mansoni*. Furthermore, we provide comparisons where possible with other schistosome snail hosts for which genome information is available, as part of an ongoing process to develop more extensive genome resources that will aid in answering the many daunting questions, we have posed above.

## Methods

### Ethics statement

This project was undertaken with the approval of Kenya’s National Commission for Science, Technology and Innovation (permit number NACOSTI/P/15/9609/4270), National Environment Management Authority (NEMA/AGR/46/2014), and snails were exported with the approval of the Kenya Wildlife Service permit numbers 0004754 and 0001680.

### *B*. *pfeifferi* snail sampling, breeding history

In 2017, *B*. *pfeifferi* specimens were collected along the vegetated margins of Kasabong Stream (-0.15190, 34.33550) and Asao Stream, Kenya (-0.31810, 35.00690). Snails were brought to the Center for Global Health Research, Kisian, Kenya, where they were checked for trematode parasites, and uninfected snails were pooled and used to establish a colony. Survivors were subsequently transported to and maintained at the University of New Mexico in 20 L plastic containers filled with 5 L of artificial spring water with high aeration using airstones. A Weco Wonder Shell was placed in snail tanks monthly for additional calcium. Snails were fed twice weekly with veggie sticks and snail growth sticks, AquaticBlendedFoods Super Freshwater Snail Mix #2. Plastic plants were added to the snail containers to allow the snails more surface area for egg laying. Algae were allowed to grow on the plastic sides of the snail tanks, and the water was changed every 1–2 months. Snails were maintained on a 12:12 light-dark cycle (25–27°C).

### *B*. *pfeifferi* snail extraction prep and whole genome sequencing

One *B*. *pfeifferi* (8 mm shell diameter) was crushed with shell in liquid nitrogen. The crushed snail was moved to a tube containing 5 ml of 30 mM Tris, 10 mM EDTA, and 1% SDS (pre-dissolved in 50 ml of water). Proteinase K (50 μl, Thermo Fisher Scientific, Waltham, MA) was added and the solution was placed in 55°C water bath for an hour. DNA was precipitated from the SDS solution with 2x ethanol. Precipitated DNA was centrifuged at 4500 x g and resuspended in 5 ml of QIAGEN G2 solution (QIAGEN, Hilden, Germany). Another 50 μl of Proteinase K was added. Once the precipitated DNA was fully dissolved in G2 solution, a Qubit assay (Thermo Fisher Scientific, Waltham, WA) was used to measure DNA concentration and the sample was loaded onto a Qiagen genomic tip column and the Qiagen Genomic DNA Preparation protocol was followed. Once the DNA was eluted from the column it was precipitated with 2x ethanol. The precipitated DNA was resuspended and dissolved in 500 μl of Elution Buffer. The Qubit assay indicated a DNA yield of 33.8 ng/μl. Genomic DNA was sheared using the Diagenode Megaruptor 2 (DNA LINK, Inc., South Korea) and checked on an Advanced Analytical Fragment Analyzer (Agilent) to determine the size of the sheared DNA. Once sheared, the sample was cleaned using 0.5x AMPure beads (Beckman Coulter, Brea, CA) and resuspended in 90 μl of PacBio EB buffer. A Qubit assay then measured 140 ng/μl of DNA. DNA (10 μg) was used in PacBio’s HiFi library preparation protocol (Procedure & Checklist—Preparing SMRTbell Libraries for HiFi Long Read Sequencing on Sequel and Sequel II Systems (Pacific Biosciences, Menlo Park, CA), Version 02 (May 2019)), then the sample was 1x AMPure cleaned, and 4 μg of DNA was loaded on the SageElf (ACCELA). The top 4 elution fractions from the SageElf were run on the Fragment Analyzer. The best fraction of DNA was from well 3. A final clean-up was applied with a 0.5x AMPure cleanup and eluted in 12 μl of EB buffer before proceeding to the binding and annealing step.

The prepared DNA sample was loaded in a PacBio Sequel II–SMRT Cell instrument (Pacific Biosciences, Menlo Park, CA) for whole genome sequencing. HiFi reads centered at 15–20 kb were obtained after 30 hours of sequencing.

### *B*. *pfeifferi* genome size analysis

Measurement of nuclear DNA content (2C) was performed at Lifeasible, Shirley, NY (www.lifeasible.com). *B*. *pfeifferi*, *B*. *glabrata* BB02 and iM line were investigated. In each species or strain, 3 individual snails were used separately for the measurement. The garden plant hosta (*Hosta plantaginea*) (Praying Hands’ 2-2-2*)* was used as control DNA. The hosta DNA was calibrated using tomato *Solanum lycopersicum*, in which the seeds were collected from Ohio State University and the DNA content (2C) was determined to be 2.02 Gb [29,30]. After calibration, the amount of DNA (2C) of the hosta was 23.33 Gb. The reason we did not use tomato as the control is our preliminary study revealed that genome size of tomato is close to that of the snails, which will lead to overlap of the fluorescence signals between hosta and snail samples. Briefly, after removing shell and ovotestis from a live snail, somatic tissues were chopped using a double-edged razor blade and filtered with a 50 μm CellTrics filter (www.wolflabs.co.uk). After extraction and staining of nuclei, the measurement was conducted in a BD Accuri C6 cytometer (BD Biosciences), using 20-mW laser illumination at 488 NM, FL-2 585/40-nm bandpass filter. Tested snail DNA content = hosta DNA content x fluorescence intensity of the tested snail / fluorescence intensity of control hosta.

Genome size estimations by *K-mer* analysis were performed using GenomeScope 2.0 [31], and *K-mer* plots were created using Merqury [32] with PacBio HiFi reads (for *B*. *pfeifferi*) and Illumina paired end reads (BB02) downloaded from NCBI sequence Read Archive (SRA) PRJNA12879 [12].

### *B*. *pfeifferi* genome assembly

Initial genome assembly was performed on PacBio HiFi subreads using Hifiasm 0.16.1-r375 [33] with default settings. Genome assembly quality assessments were performed for initial and final genome assembly using assembly-stats v1.0.1 (https://github.com/sanger-pathogens/assembly-stats), Benchmarking Universal Single-Copy Ortholog assessment tool BUSCO 4.1.4 [34], and QUAST v5.0.2 [35].

The genome assembly was cleaned and filtered based on supportive read depth, GC content, and sequence similarity search results using BLAST [36] and DIAMOND [37] and visualized using BlobTools v1.1.1 [38]. The assembled contig sequences were first split into 10 kb fragments, which were submitted as queries to search against NCBI non-redundant nucleic acid (NT) and protein databases (NR) [39] with corresponding options: for BLASTn against NT “-max_target_seqs 20 -max_hsps 1 -evalue 1e-5 -perc_identity 60”; for DIAMOND BLASTx to NR “-f 100—max-target-seqs 20—masking 1—tmpdir /tmp/—evalue 1e-5—salltitles -b 60.0”. Taxonomy classification at phylum level, contig coverage, and GC content of contig sequences were collected and visualized in Blobplot. Contig sequences with top blast hit to Acidobacteria, Bacteria-undef, Bacteroidetes, Microsporidia, Nitrospirae, Proteobacteria, Verrucomicrobia, Viruses-undef were filtered. Contigs with no BLAST hits or with Mollusca hits were kept. Contigs were sorted from longest to shortest and renamed in that order.

### *B*. *pfeifferi* genome annotation

**Fig 1:**
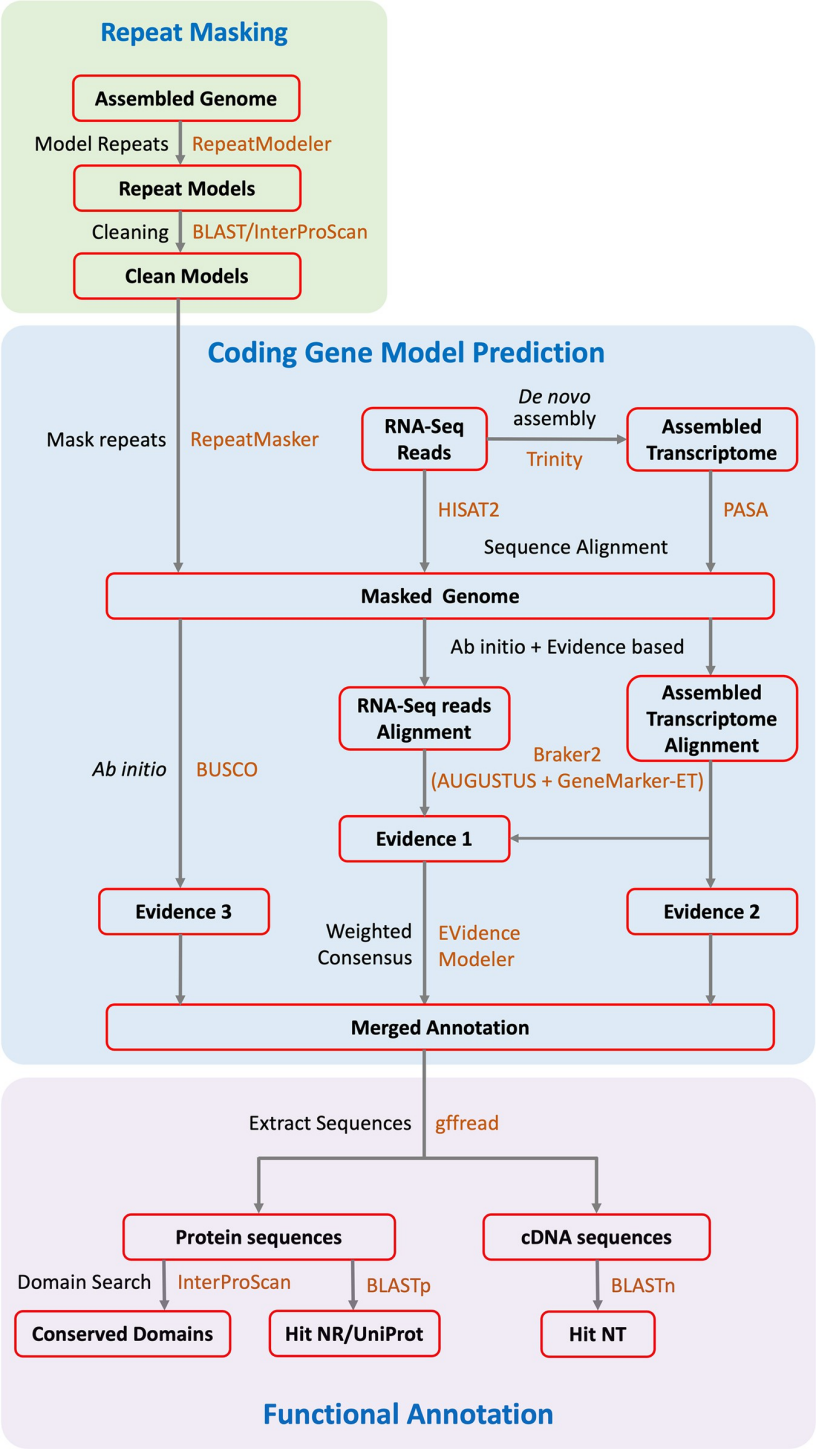
A typical eukaryote genome annotation workflow of protein coding gene annotation for the *B*. *pfeifferi* genome. The workflow contains three stages (boxes): repeat-masking (green), gene model prediction (blue) and functional annotation (purple). The intermediate and final results were labeled in boxes with red frames. The analytical processes were marked out in black-colored text and bioinformatics tools marked in orange-colored text.

A typical eukaryote genome annotation workflow contains three stages: repeat masking, gene model prediction, and functional annotation (Fig 1). We started genome annotation with repetitive elements which are known to occupy more than 40% of snail genomes [12,16,40]. Repeat sequence models were built with a cleaned genome assembly using RepeatModeler 2.0.1 [41] in Dfam transposable elements TE Tools v1.2 [42]. Genes from large gene families could be mistakenly interpreted as repeats during the repeat modeling. Therefore, the predicted repeat models were searched against InterProScan database 5.45 [43], to retain models with either no domain or only retrotransposon domains and to exclude any other functional domains. Finally, the clean genome assembly was soft masked (repeats in lower-case letters) with clean repeat models using RepeatMasker 4.062 [44].

Gene models were predicted using EVidence Modeler 06/25/2012 [45], which incorporates weighted evidence from *ab initio* predictions, RNA sequencing (RNA-Seq) alignments, and sequence similarity-based searches against known transcript sequences.

The *ab initio* prediction evidence is comprised of core single copy orthologs identified using BUSCO customized HMM models built from tBLASTn [36] and AUGUSTUS predictions [46]. Evidence from spliced alignments of expressed transcript sequences was generated by mapping *de novo* assembled *B*. *pfeifferi* transcripts [27] to the cleaned masked genome assembly, using the “Program to Assemble Spliced Alignments” (PASA) pipeline [47].

RNA sequence evidence was generated from genome mapping of RNA-Seq data (NCBI PRJNA383396) from *B*. *pfeifferi* schistosome infection studies [27,28,48] using HISAT2 version 2.2.1 [49]. A total of 1,186,204 transcripts were obtained from the RNA-Seq study by Buddenborg et al., 2017 [27]. Transcripts sequences were cleaned up in the PASA pipeline, to obtain non-redundant coding transcripts using SeqClean and Transdecoder, and 158,095 clean sequences were generated. PASA utilized two alignment methods to align 158,095 clean transcripts to the *B*. *pfeiffer*i genome. The mapping rates are 82.6% for BLAT (130518/158,095) and 83.0% for GMAP (131232/158,095). Raw RNA-Seq sequences were first downloaded from the NCBI Sequence Read Archive (SRA) using the prefetch program, then converted to FASTQ format using the fastq-dump program in SRA toolkit 2.9.6 (https://trace.ncbi.nlm.nih.gov/Traces/sra/sra.cgi?view=software). Low quality sequences in FASTQ reads were filtered and trimmed using Trimmomatic 0.36 [50]. A sliding window of 4 bases was used to identify and remove the part of reads where average quality dropped below a score of 20. For samples recovered from snails infected with *S*. *mansoni*, schistosome sequences were filtered out by mapping cleaned RNA-Seq reads to *Schistosoma mansoni* Puerto Rico strain genome V7 PRJEA36577 obtained from WormBase Parasite (WormBase web site, http://www.wormbase.org, release WBPS15, date Mar. 20, 2021). Clean reads were mapped to the cleaned, repeats-masked *B*. *pfeifferi* genome assembly using HISAT2. The alignments of RNA-Seq reads and assembled transcripts were used as input for the BRAKER 2.1.1 annotation pipeline [51], in which GeneMarker-ET [52] was used to generate hints to train AUGUSTUS separately, then merged training as described in the BRAKER2 pipeline was followed.

With the evidence described above, EVidence Modeler was used to predict gene models. BRAKER2 evidence was given weight 10 and all other evidence weight 1 based on BUSCO reports of source evidence and results of different weights combinations. The predicted gene models were sorted, renamed based on contig order and gene start location. Transcript, coding and protein sequences were extracted using GffRead 0.9.12 [53] with options “-V -H -B -w transcripts.fna -x cds.fna -y protein.faa -g../genome.fasta”.

Functional annotation for predicted gene models was performed by sequence similarity searches. Extracted coding sequences and protein sequences were searched as queries against reference databases: 1) BLASTp to UniProt [54] database with minimum identity of 30%, minimum aligned length 10 aa, and max E value 10^−5^; 2) BLASTp to the NCBI non-redundant protein database (NR) with the same cut off as above; 3) BLASTn to the NCBI non-redundant nucleotide database (NT) with minimum identity of 60% and maximum E value 10^−5^; 4) conserved functional domain by InterProScan 5.45 [43]. *Biomphalaria* specific IgSF domains were predicted by a *B*. *glabrata* specific HMM model [55] with minimum length of 40 aa, and max E value of 0.00165.

### *B*. *pfeifferi* RNA prediction (tRNA, rRNA, microRNA, long-noncoding RNA)

Transfer RNAs (tRNAs) were predicted using tRNAscan-SE 2.0.9 [56] with standard parameters. Ribosomal RNAs (rRNAs) were predicted using Barrnap 0.9 (https://github.com/tseemann/barrnap) with options “—kingdom euk—threads 40”.

For microRNAs (miRNAs), conserved RNA sequence similarity sharing sequence and/or structural similarities within the genome were identified. Sequence profiles containing covariance models (CMs) of known non-coding RNAs were downloaded from the Rfam database, and used as queries to search within the assembled genome using Infernal (INFERence of RNA ALignment) software [57], with options “cmscan—cpu 40—rfam—cut_ga—nohmmonly—tblout mrum-genome.tblout—fmt 2—clanin Rfam.clanin Rfam.cm”. miRNA sequence similarity identified in the *B*. *pfeifferi* genome were extracted. Additionally, a highly automated pipeline with classified miRNA CMs, named mirMachine pipeline 0.2.11.2, was used to find miRNAs with the node set to Mollusca. The miRNAs predicted from the two methods were merged and duplicated records with overlapping location were removed. miRNAs in *B*. *pfeifferi* and *B*. *glabrata* were grouped using cd-hit-est 4.8.1 [58].

The annotation of long non-coding RNAs (lncRNAs) was initiated by mapping known RNA-Seq reads to the clean *B*. *pfeifferi* genome assembly. The genome-guided transcriptome assembly was based on the RNA-Seq read-to-genome alignment using Trinity 2.8.5 [59]. Assembled transcripts were filtered, and only sequences longer than 200 nt (length in nucleotides) were kept. The sequence coding potential of each transcript sequence was checked by Coding Potential Calculator version 2 (CPC2) [60]. Possible coding protein sequences were further filtered by BLASTx search against the NCBI NR database with options “-f 100—max-target-seqs 20—masking 1—evalue 1e-5—salltitles -b 60.0”. Other ncRNAs were filtered based on hits to Rfam CMs using Infernal (INFERence of RNA ALignment) tools described above. Finally, clean RNA-Seq reads were mapped to transcripts, and only transcripts with transcripts per million (TPM) values between 3 and 2000 were kept.

### Comparison of overall genomic structures of *B*. *glabrata* and *B*. *pfeifferi*

To facilitate a whole genome alignment, the cleaned contig sequences of the *B*. *pfeifferi* genome assembly were aligned to the *B*. *glabrata* iM line genome sequences using minimap2 program [61] with options “-ax asm5—eqx”. Genomic differences, including syntenic path (longest set of co-linear regions), structural rearrangements (inversions, translocations, and duplications), local variations (SNPs, indels, CNVs etc) within syntenic and structural rearrangements, and un-aligned regions were predicted using the comprehensive tool SyRI [62] with suggested options “-c out.sam -r refgenome -q qrygenome -k -F S”. Visualization of structural variations (SV) were plotted using ggplot2 v3.1.0 [63] and ShinySyn [64] in the R environment [65].

### Inferred linkage groups for *B*. *pfeifferi* derived from synteny comparisons with *B*. *glabrata* linkage groups

Using the whole genome alignment information generated above, the *B*. *pfeifferi* contigs that best matched to *B*. *glabrata* iM line with the largest non-redundant scaffold’s coverage were identified. These best-matched *B*. *pfeifferi* contigs were sorted according to iM line and scaffolds per iM line’s 18 linkage groups. Inferred linkage groups (18) were assigned to *B*. *pfeifferi* contigs according to their best matched iM line linkage group. Data transformations were processed using in house Linux scripts and R tidyverse package [66].

### Split time estimation

Protein sequences were obtained from NCBI for species with annotated genome assemblies: *B*. *glabrata* iM line (International Nucleotide Sequence Database Collaboration (INSDC) genome ID JAKZJL000000000), *Bu*. *truncatus* (JAGDYQ000000000), and *Elysia marginata* (BMAT00000000). For out-group *Radix auricularia* (MUZC00000000), no annotated genes in NCBI were available, so the protein sequences for core Mollusca orthologs were predicted from the genome sequences using BUSCO.

With all protein sequences, orthologs were identified using OrthoFinder 2.5.4 [67], and a species tree was generated using algorithm Species Tree from All Genes (STAG) [68], with tolerance for few or no complete sets of one-to-one orthologs present in all species.

The timetree was inferred by applying the RelTime method [69,70] to the estimated species tree with branch lengths in MEGA X [71]. The split time of *B*. *pfeifferi* and *B*. *glabrata* was calculated based on one fixed calibration constraint on the time tree: the appearance of *Bulinus* in the fossil record at least 19–20 Mya [72].

### Gene families of interest in the *B*. *pfeifferi* genome

Based on the predicted gene models with InterProScan, UniProt, DeepTMHMM [73] in the *B*. *pfeifferi* genome, we further classified and analyzed a list of immune response-related gene families of interest, including fibrinogen-related domain-containing proteins (FReDs) [12,55,74–78], C-type lectin-related proteins (CREPs) [79,80], and galectin-related proteins (GREPs) [79], AIG (avrRpt2-induced gene) family of GTPases (AIGs) [81], biomphalysins [82,83], G-protein coupled receptors (GPCRs) [12] and Toll-like receptors (TLRs) [12,84,85]. We also searched for chemosensory communication pheromones, including the four described in the *Aplysia californica*/*brasiliana* pheromonal complex, attractin, seductin, enticin, and temptin [86,87].

Basic gene family classification was based on sequence similarity search using the BLASTn or BLASTp program mentioned above against NCBI database, and Uniprot databases with a minimum of 85% identify and coverage. Higher level gene family classification was based on conservative domain structures, e.g., fibrinogen-related proteins (FREP) containing immunoglobulin superfamily (IgSF) and fibrinogen (FBG) domains [12,55]; AIGs/GIMAPs containing an AIG1 domain [73,81]; biomphalysins containing an aerolysin domain, and pore-forming transmembrane domains (TMDs) predicted with the PRED-TMBB server (PREDiction of TransMembrane Beta Barrels proteins) [88,89]; HSP70s were characterized by the conserved HSPA -nucleotide-binding domain (HSPA1-A, -B, -L, HSAP-2, -6, -7, -8), and similar proteins (cd10233) and nucleotide-binding domain of the sugar kinase/HSP70/actin superfamily (cd17037) [12]; C-type lectin-related proteins (CREP) containing C-type lectin/C-type lectin-like domains (CLECT domain) [80]; galectin-related proteins (GREP) containing galectin domains (GLECT domain) [80]; and conservative Ca^2+^ binding EGF-like domain, DSDXD in temptin [87,90].

### Maximum likelihood tree of *B*. *pfeifferi* FReDs

Full-length protein sequences of all identified *B*. *pfeifferi* FReDs in this study were used to construct a maximum likelihood tree through IQ-TREE [91] with standard model selection followed by 1000 bootstrap replicates. The best substitution model was WAG+F+I+G4 chosen according to a minimal Bayesian information criterion (BIC) score using ModelFinder tool [92] in IQ-TREE.

### *B*. *pfeifferi* FReDs nomenclature

**Fig 2:**
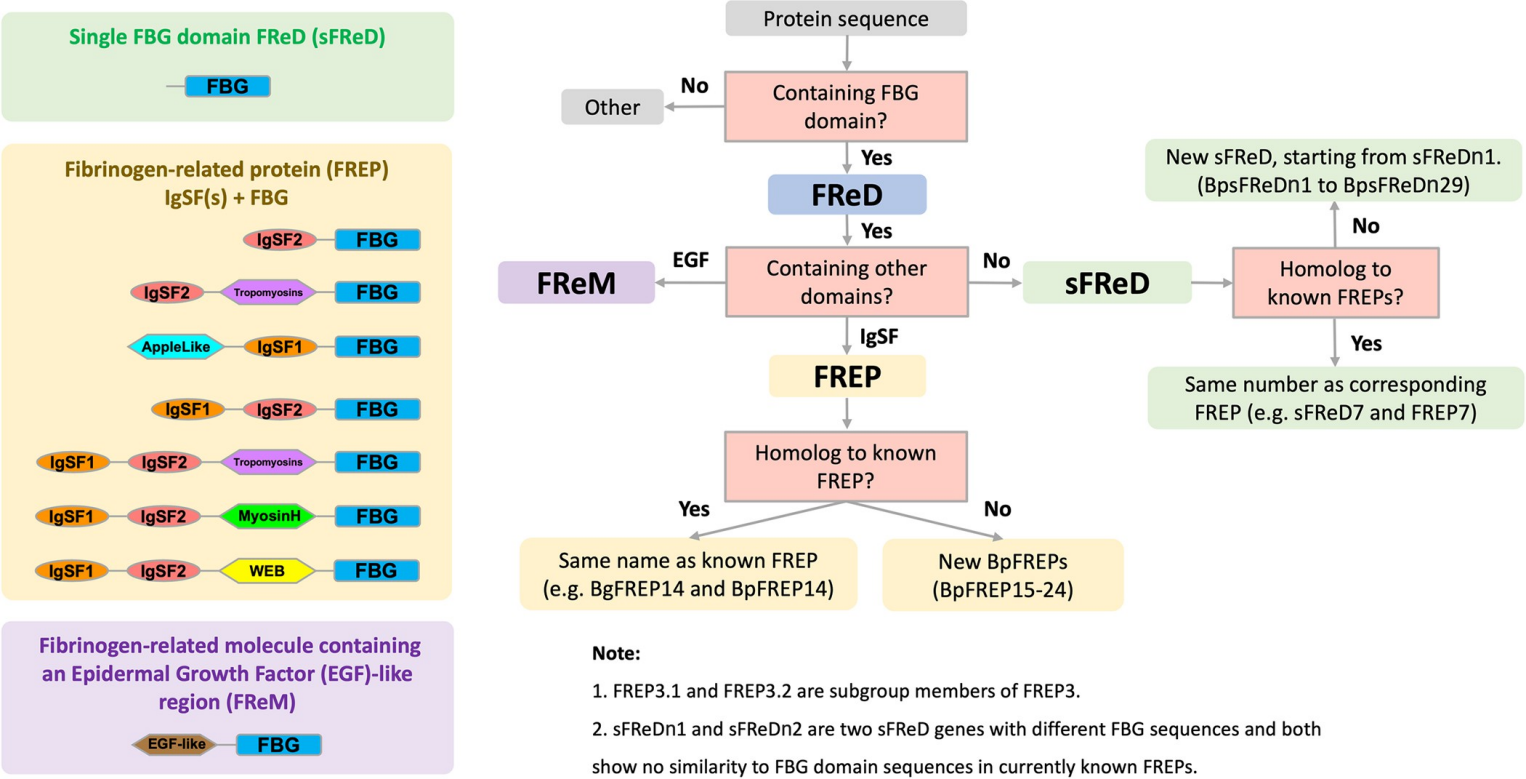
Nomenclature for *B*. *pfeifferi* Fibrinogen-Related Domain-containing proteins (FReDs). The FReD family is an inclusive, more general group containing the following subgroups: 1) sFReDs, or single FBG FReDs (containing FBG domain only); 2) FREPs, with 1 or 2 IgSF(s) joined via an interceding region (ICR) to a single FBG domain; and 3) FReMs (consisting of an epidermal growth factor domain (EGF) and a FBG domain. FREPs are therefore a sub-category within the FReD family. The domain structures for three main classes of known FReDs (green for sFReDs, yellow for FREPs and purple for FReMs) are summarized on the left, edited from Lu et. al (2020). The nomenclature logic process was displayed as a decision tree on the right. Naming convention tests (yes, no, or other) were made based on the test questions in pink boxes with gray frames. Final names were assigned into 5 classes: FReM, FREP1-14, FREP15-24 (novel FREPs), sFReD1-14, and sFReDn1-n29 (new sFReDs).

A systematic nomenclature is presented in this study (Fig 2) for the *B*. *pfeifferi* fibrinogen-related domain-containing proteins (FReDs) family, one that complements and does not conflict with the nomenclature developed for the *B*. *glabrata* FReDs family. This is important because of the immunological role of FREPs (one of the core groups of FReDs) in host responses in *B*. *glabrata* to parasites [76,93,94], and because it is a complex gene family, with both familiar and new FReDs identified in *B*. *pfeifferi*.

*B*. *pfeifferi* FREPs were assigned based on IgSF and FBG domain verification and the presence of a signal peptide. FREP1 to FREP14 have been reported in *B*. *glabrata* studies [12,55,95,96]. Following the same naming system, *B*. *pfeifferi* sequence similarity BpFREP1 to BpFREP14 were identified (≥ 85% sequence identity at nucleotide level) to known *B*. *glabrata* FREPs. Additionally, for some BpFREPs, an additional numeral was added to distinguish individual variants (sequence similarity between 85% to 100%), e.g., BpFREP12.1, 12.2, and so on. Several BpFREPs had no obvious similarity to the known FREPs in the *B*. *glabrata* genome. Such new BpFREPs were named starting with BpFREP15, and so on.

Naming of *B*. *pfeifferi* sFReDs (single Fibrinogen-Related Domain-containing proteins) and FReMs (fibrinogen-related molecules containing an epidermal growth factor (EGF)-like region) was also made in reference to sequence similarity to the fibrinogen domains in *B*. *pfeifferi* FREPs; sFReDs and FReMs were assigned names following the BpFREPs with the closest fibrinogen sequence. For instance, BpsFReD12.1 shows closest similarity to the fibrinogen domain in BpFREP12.1, and BpsFReD12.4 shows high sequence similarity to the fibrinogen domain in BpFREP12.4. For *B*. *pfeifferi* sFReDs that have no similarity to any known FReDs [55], they were named as “new” sFReD, with “n” representing “new”, with format BpsFReDn1, BpsFReDn2, and so on. Similar nomenclature was applied for *B*. *pfeifferi* FReMs, e.g. BpFReM1, BpFReM2, and so on.

### Allelic variation analysis in several FREPs among *B*. *pfeifferi* and *B*. *glabrata* strains

Several single nucleotide variants (SNV) have been identified among BgFREP 2,3, and 4 derived from different strains or isolates of *B*. *glabrata* (BB02, BS90 and M line), including strains varying in susceptibility to *S*. *mansoni* [55]. It was of interest to include similar FREPs from *B*. *pfeifferi* in such comparisons. Therefore, BpFREP 2, 3, and 4 transcript sequences were aligned and blasted against *B*. *glabrata* sequence similarity. For each FREP gene of interest, protein sequences from the *B*. *glabrata* BB02 genome [12], similar *B*. *glabrata* BS90 and M line strain transcript sequences [55,97], and similar *B*. *pfeifferi* transcript sequences (this study) were aligned together using the multiple sequence alignment program MAFFT [98] and visualized using JalView 2.11.0 [99].

### Alphafold2 analysis of several *B*. *pfeifferi* FREPs

The three-dimensional (3D) structures of selected *B*. *pfeifferi* FREPs were predicted based on the protein sequences using the Alphafold2 algorithm [100] integrated in ColabFold software [101], a platform with optimization to speed up the sequence search and the prediction process. The protein sequences of identified FREPs were submitted via command colabfold_batch with options “—amber—templates—num-recycle 3—num-models 1 input.fasta outdir—cpu”. The predicted Protein Data Bank (PDB) format files were visualized using the molecular graphics program Visual Molecular Dynamics (VMD) [102]. To provide some comparison, the protein sequence of BgFREPs from the *B*. *glabrata* genome was submitted and processed with the same Alphafold2 analysis.

### Sequence similarity in the *B*. *pfeifferi* genome for possible schistosome resistance-related genes identified in previous genome-wide association studies in the *B*. *glabrata* genome

Several *B*. *glabrata* genome-wide association studies (GWAS) have been undertaken to identify candidate genes in particular chromosome regions associated with resistance (or susceptibility) to infection with *S*. *mansoni* [40,103–106]. It is of interest to know if sequence similarity of the identified GWAS genes, and the relative positions and composition of the resistance complexes identified in *B*. *glabrata*, can be found in the *B*. *pfeifferi* genome. The gene sequences from the previous GWAS studies were extracted [97] and were used as subjects to be BLASTed against, to obtain the closest sequence similarity in *B*. *pfeifferi*, with query sequence coverage and identity ≥85% as a cutoff value.

### Other analyses

Signal peptide searches were performed using SignalP 4.0 [107]. Venn diagrams were generated using R package VennDiagram v1.6.20. Summarized tables and figures were generated using R base packages [65] and ggplot2 v3.1.0 [63].

## Results

### *B*. *pfeifferi* genome assembly

The *B*. *pfeifferi* haploid genome size assembled from PacBio HiFi reads is 772 Mb, smaller than observed for other related schistosome vector snails, including two other species of *Biomphalaria* and *Bu*. *truncatus* (Table 2). The estimated genome size for the Brazilian field-derived *B*. *glabrata* BB02 is 916 Mb, this being the first schistosome vector snail genome obtained [12]. Because the BB02 genome was largely pieced together using different methods, it is not included in this table; its genome size is comparable to two *B*. *glabrata* representatives sequenced more recently, iM line and iBS90, the former shown in Table 2. The summary of complete and fragmented BUSCOs of *B*. *pfeifferi* and related snail genomes is in S1 Table.

**Table 2:** Summary statistics of the genome assembly of *B*. *pfeifferi* and related snail genomes.

Genome assembly	*B*. *pfeifferi*	*B*. *glabrata* iM line	*B*. *straminea*	*Bu*. *truncatus*
NGS Platform	-	Illumina PE	Illumina PE	Illumina PE
Number of reads	-	410,797,214	-	-
Mean of read (bp)	-	150 x 2	150 x 2	150 x 2
Median of read (bp)	-	150 x 2	150 x 2	150 x 2
Total of bases (Mbp)	-	123,239	85,030	128,500
Coverage	-	149x	68x	100x
TGS Platform	PacBio CCS (HiFi)	PacBio CLR	-	-
Number of reads	1,543,626	7,308,853	-	-
Mean of read (bp)	18,399	13,712	-	-
Median of read (bp)	18,366	24,502	-	-
Total of bases (Mbp)	28,401	100,217	-	-
Coverage	37x	120x	-	-
**Assembly Statistics**				
Total Length (bp)	771,836,736	870,959,050	1,004,745,081	1,221,776,979
Number of contigs	505	255	84,585	523
Mean contig length (bp)	1,528,390	3,415,526	11,879	2,336,094
Longest contig length (bp)	14,637,294	60,252,455	58,377,830	36,501,513
Shortest contig length (bp)	18,051	1,308	938	10,033
Contig N50	3,253,497	22,698,051	25,272,813	4,956,851
Contig L50	72	13	15	68
Number of contigs (> = 10 Kbp)	505	238	9,057	523
Total length (> = 10 Kbp)	771,836,736	870,886,136	755,905,799	1,221,776,979
GC content	36%	36%	36%	36%
BUSCO*	96%	96%	87%	96%

Note

*Complete and fragmented BUSCOs found from OrthoDB v10 Metazoa datasets (N = 954, https://busco.ezlab.org/list_of_lineages.html) (see S1 Table). Abbreviations: NGS, Next Generation Sequencing; PE, paired end; TGS, Third Generation Sequencing; CCS, Circular Consensus Sequence; HiFi, High-Fidelity; CLR, Continuous Long Reads. The three *B*. *glabrata* genomes in the study by Tennessen et al. (2020) [106] were not listed in this table due to lack of genome annotation.

An independent estimation of genome size based on fluorescence cell sorting intensity in comparison with known standards (Lifeasible) yielded a value of 913 Mb for *B*. *pfeifferi*, again notably smaller than values obtained for *B*. *glabrata* BB02 (1,020 Mb) or *B*. *glabrata* iM line (1,090 Mb) using the same technique (Table 3). The consistently higher values obtained from fluorescent cell sorting relative to those from sequencing approaches is probably a consequence of the latter technique underestimating the amount of repetitive DNA [108].

**Table 3:** Estimation of genome size based on fluorescence cell sorting intensity in comparison with known standards.

Name	Sample	Raw 2C^a^	Estimation (Mb)	Average (Mb)
*B*. *pfeifferi*	BiPf-1	1.87	935	913
	BiPf-2	1.87	935	
	BiPf-3	1.74	870	
*B*. *glabrata* iM line	iM line-1	2.26	1,130	1,090
	iM line-2	2.17	1,085	
	iM line-3	2.11	1,055	
*B*. *glabrata* BB02	BB02-1	2.11	1,055	1,020
	BB02-2	2.04	1,020	
	BB02-3	1.97	985	

Note

^a^: Raw genome size (2C, C is defined as the mass of DNA present in a haploid chromosome set) estimated based on fluorescence cell sorting intensity in comparison with known standards.

### The *B*. *pfeifferi* genome is relatively homozygous

**Fig 3:**
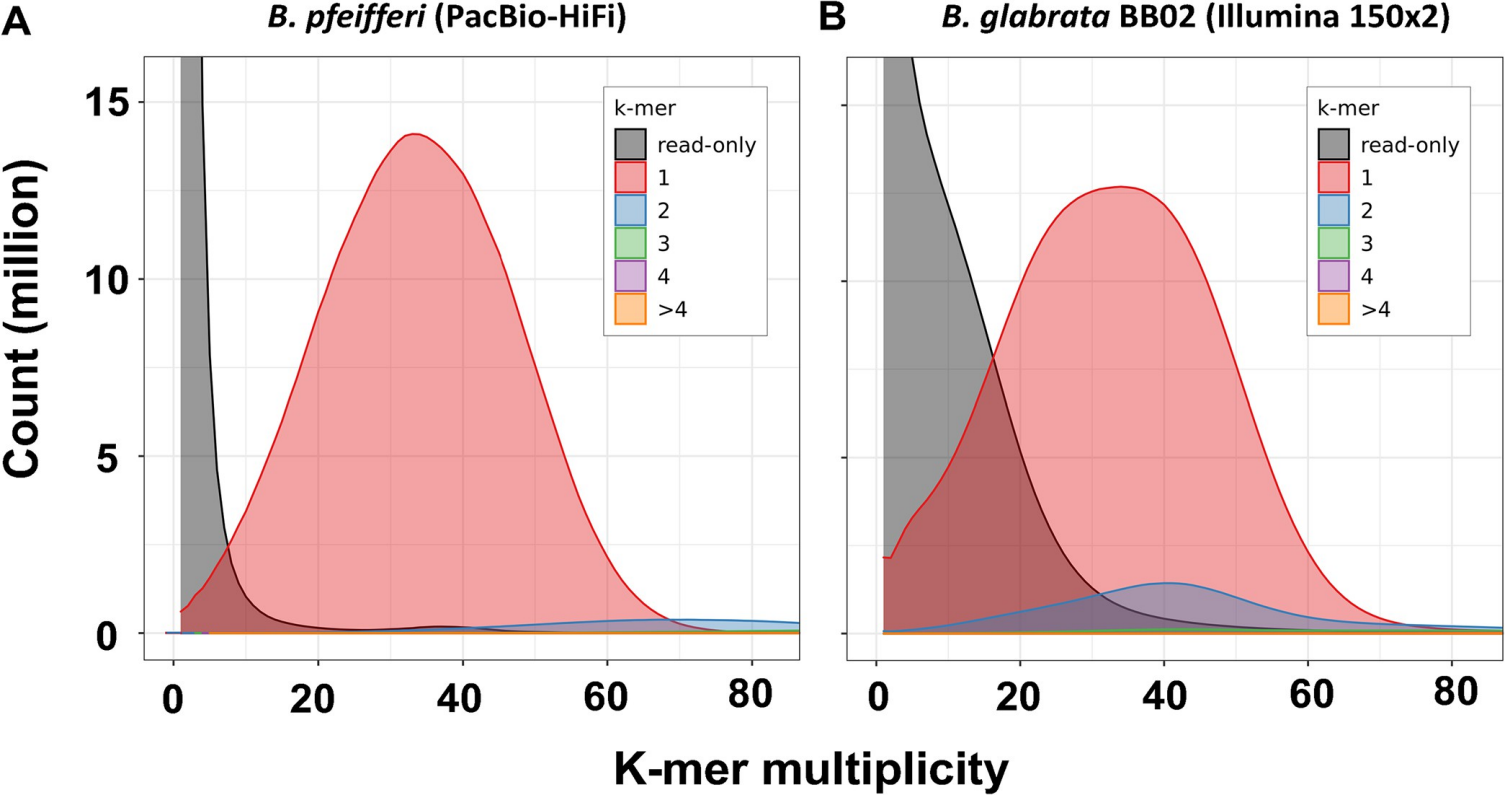
Merqury copy number spectrum plots show low heterozygosity of the *B*. *pfeifferi* genome compared to the *B*. *glabrata* BB02 genome. Histograms were colored by copy numbers: k-mers present in reads but missing in genome assembly were in grey; k-mers present in both raw reads and genome assemblies were colored in red (1x), blue (2x), green (3x), purple (4x) and orange (>4x). A single peak (red) was found in (A) PacBio HiFi reads for *B*. *pfeifferi*, whereas both a red and blue peak were obtained in (B), the Illumina paired end reads for *B*. *glabrata*.

This observation is supported by three lines of evidence. First, the relatively high peak of the 1-copy k-mer curve and the limited area under the 2-copy k-mer peak (see blue) in *B*. *pfeifferi* are indicative of a homozygous genome [109] (Fig 3A). By comparison, the more extensive 2-copy k-mers peak (blue) in *B*. *glabrata* is indicative of a greater degree of heterozygosity (Fig 3B).

Secondly, k-mer analysis using GenomeScope showed the *B*. *pfeifferi* genome heterozygosity rate to be 1.01% (PacBio HiFi reads), which is lower than 1.52% for outbred *B*. *glabrata* BB02. For comparisons sake, the deliberately inbred iM line with a history of 70+ generations of forced selfing had a heterozygosity rate of 0.197% [40].

The third line of evidence of high homozygosity in the *B*. *pfeifferi* genome derives from RNA-Seq data (S1 Fig). The percentage of homozygous SNPs found in field-derived *B*. *pfeifferi* is about 80%. Remarkably, this is higher than the 60% value for homozygous SNPs identified from the *B*. *glabrata* M line strain which, though originally derived from a cross between Brazilian and Puerto Rican snails, has been maintained in laboratory colonies for nearly seven decades [110].

### Genome annotation: coding gene model prediction and functional annotation

**Fig 4:**
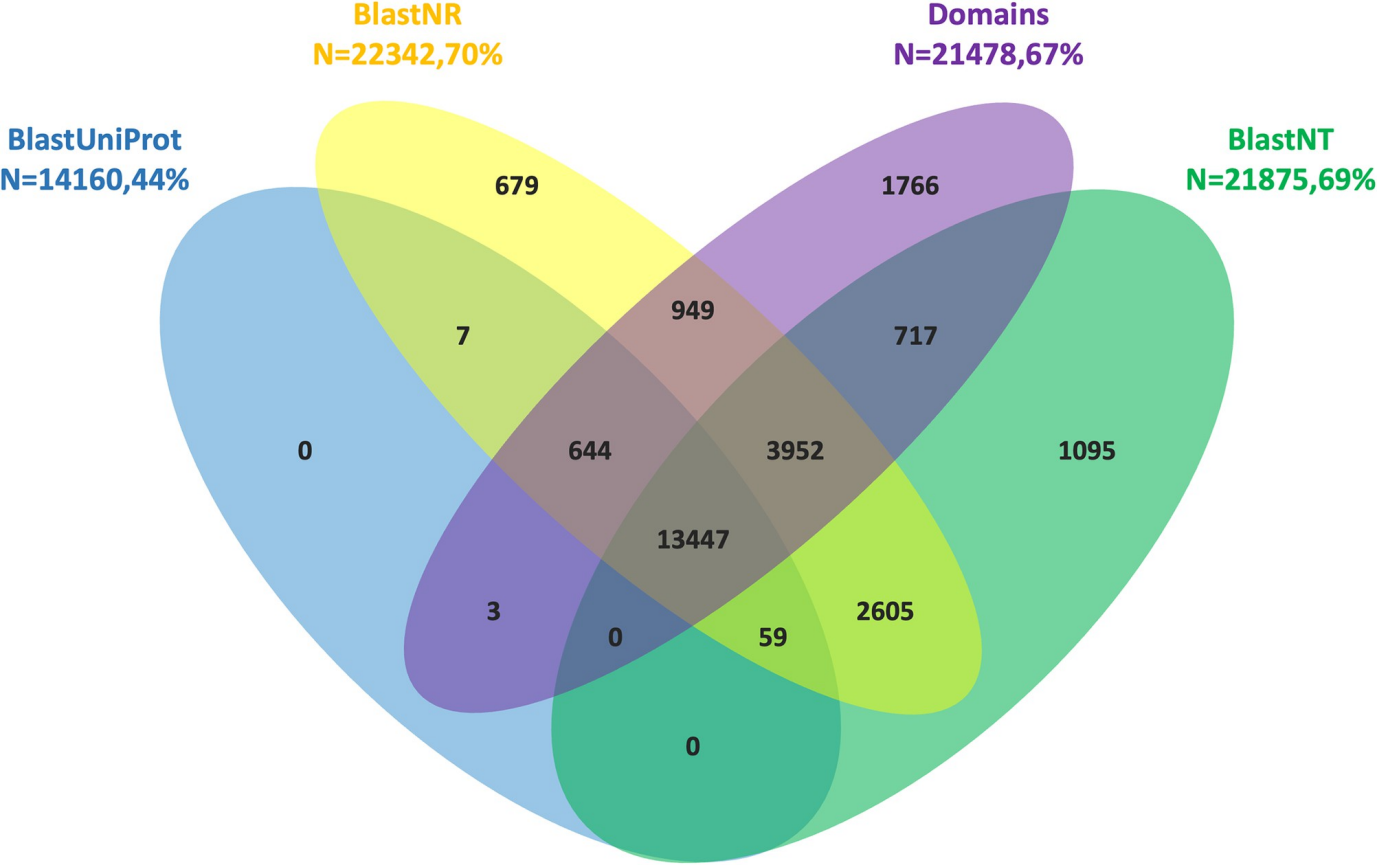
Functional annotation for protein coding gene models predicted in the *B*. *pfeifferi* genome.

A total of 31,894 protein-coding gene models were predicted for the *B*. *pfeifferi* genome. The predicted genes scored varying numbers of similarity hits against four available databases (Fig 4): 1) Uniprot manually curated high-quality protein database 14,160 (44%); 2) NCBI non-redundant nucleotide NT database 21,875 (69%); 3) NCBI non-redundant protein NR database 22,342 (70%); and 4) InterProScan database 21,478 (67%), the latter being an assembly of 14 conservative protein domain databases. From the total of 31,894 gene models, 13,447 (42.16%) were annotated by all four databases and 25,923 (81.28%) were annotated from at least one of the four sources. For comparison, the recent annotation process for the *Bu*. *truncatus* genome estimated that 83.5% of protein-encoding genes had been annotated by one or more methods [16].

### Annotation of repetitive elements in the *B*. *pfeifferi* genome

The overall portion of the genome occupied by repetitive elements for *B*. *pfeifferi* is 329 Mb (S2 Table), smaller than for *B*. *glabrata* iM line (374 Mb) or *B*. *glabrata* iBS90 (391 Mb). Additionally, the proportion of the genome occupied by repetitive elements is slightly smaller in *B*. *pfeifferi* (42.73%) than in either *B*. *glabrata* strain (42.95% and 44.17%, respectively).

As noted in S2 Fig, the types of repeats less represented in *B*. *pfeifferi* are Gypsy/DIRS1 (6–17 Mb), simple repeats (5–6 Mb) and RTE/Bov-B iM line (17 Mb). In contrast, the hobo-Activator family, a type of DNA transposon, was increased in representation by 1.4–2 Mb in *B*. *pfeifferi* compared to the *B*. *glabrata* genome.

### Comparisons of *B*. *pfeifferi* to *B*. *glabrata* iM line genome

**Fig 5:**
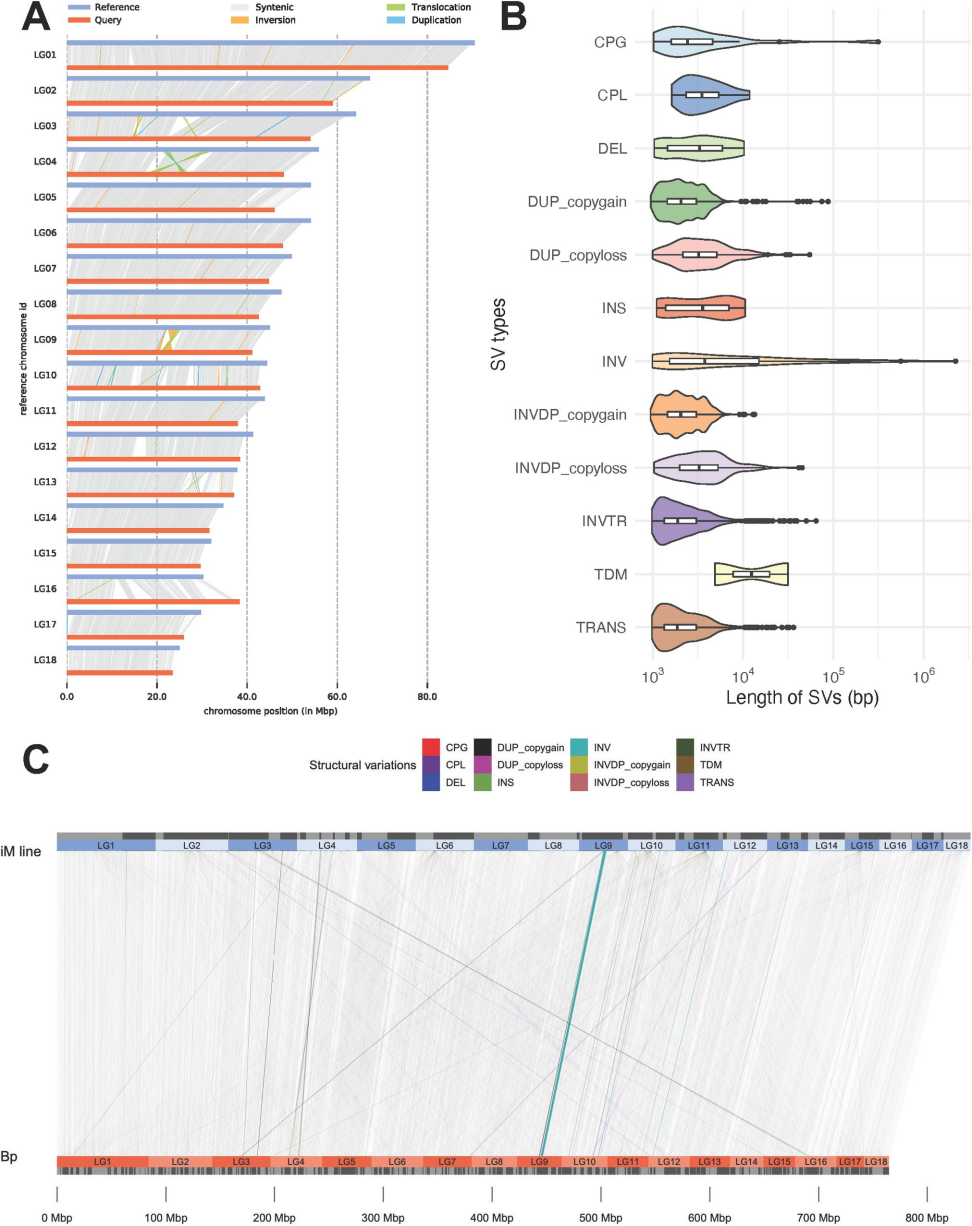
Synteny plot and structural variations (SVs) between assemblies of *B*. *pfeifferi* contigs as query matched against the 18 linkage groups of *B*. *glabrata* iM line. **A)** Parallel synteny view between *B*. *pfeifferi* (query in orange color) and *B*. *glabrata* iM line (reference in blue color). Reference and query genome were marked in blue and brown colors. Regions with synteny and structure variations detected by SyRI were highlighted in colored lines: Syntenic (grey), Inversion (orange), Translocation (green), and Duplication (blue). **B)** Length distribution in violin plots for structural variation (> 1Kb) of *B*. *pfeifferi* in comparison to *B*. *glabrata* iM line. SV types were highlighted in different colors. CPG: Copy gain in query, CPL: Copy loss in query, DEL: Deletion in query, DUP: Duplicated region, INS: Insertion in query, INV: Inverted region, INVDP: Inverted duplicated region, INVTR: Inverted translocated region, TDM: Tandem repeat, TRANS: Translocated region. Inside violin plots, box plots were placed to indicate mean, quartiles and outliers (black dots). **C)** Sequential synteny view between *B*. *pfeifferi* (query in orange color) and *B*. *glabrata* iM line (reference in blue color). Separated scaffolds inside each linkage group were marked out as grey and black boxes. Synteny blocks between two genomes are laid out in grey blocks in the background. Particularly noteworthy is the inversion indicated on LG 9.

The 18 inferred linkage groups (LGs, representing the 18 haploid chromosomes) for *B*. *pfeifferi* were determined by comparison with the 18 LGs of *B*. *glabrata* iM line, which were genetically determined using F2 offspring bred from two lines of *B*. *glabrata* [40]. Overall, 430 of the 505 scaffolds representing 99.09% of the 771,836,736 bases in the *B*. *pfeifferi* genome assembly were assigned to 18 LGs (Fig 5). The order of contigs within LGs and the detailed size of the 18 inferred linkage groups are listed in S3 Table.

**Fig 6:**
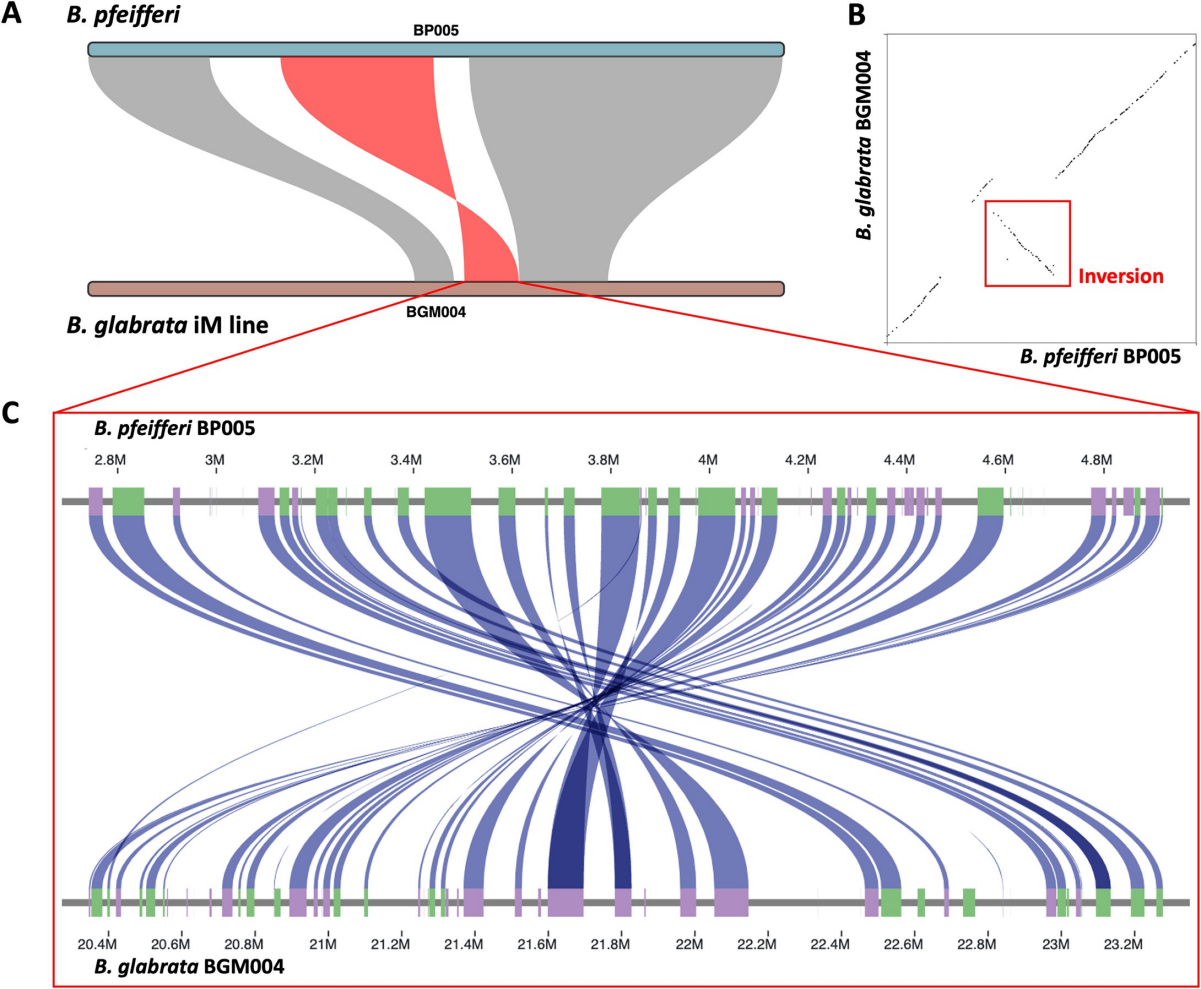
Inversion identified on inferred LG9 between *B*. *pfeifferi* and *B*. *glabrata* iM line genomes. **A**) A 2.3 Mbp size inversion region was identified. The scaffolds covering the linkage groups are BP005 and BGM004, respectively. **B**) Dot plots show the inversion area was between large flanking synteny blocks. **C**) ShinySyn plot showing the sequence similar genes connected by blue ribbons, revealing the reverse order within the inverted region. Genes were marked in colored boxes along the x axes, purple boxes for positive strand and green boxes for negative strand. Boxes without connections are genes with no sequence similarity identified between the two species.

A total of 12 types of structural variation (SV) of >1Kb were identified and summarized from a whole genome comparison between *B*. *pfeifferi* and *B*. *glabrata* iM line. These SVs occupied 8% of the *B*. *pfeifferi* genome (S4 Table). SVs had a median length of 2,051 bp, a mean of 2,997 bp, and the largest SV was an inversion of 2,259,716 bp in length. This inversion was located near the middle of LG9 (Fig 6) and consisted of 2,259,716 bp and 69 coding genes and 3 long non-coding RNA (lncRNA) in *B*. *pfeifferi*, whereas in *B*. *glabrata* iM line consisted of 1,875,396 bp and 78 protein coding genes. According to BLASTp results, 24 of these genes are reciprocal best hits. Several interesting genes locate in the inversion region, such as: inactive phospholipase C-like protein 2, putative ammonium transporter 1, serpin B3-like, syndetin-like and vinculin. They are large genes that collectively occupy 92% (650/707 Kbp) of the inversion in *B*. *pfeifferi*, and 87% (784/901 Kbp) of the comparable region in *B*. *glabrata* iM line. Further details can be found in S5 Table.

Duplicated region changes (DUP and INVDP) are the major SVs detected, accounting for 22.5 Mb (2.9%) of the *B*. *pfeifferi* genome. The duplicated regions may relate to the expansion of the FReD (fibrinogen-related domain-containing proteins) family, the size of which is increased in comparison to *B*. *glabrata*. The genes affected by the SVs would be good candidates to explore with respect to explaining the phenotypic differences between the two species.

### Split time estimation for *B*. *pfeifferi* and *B*. *glabrata*

**Fig 7:**
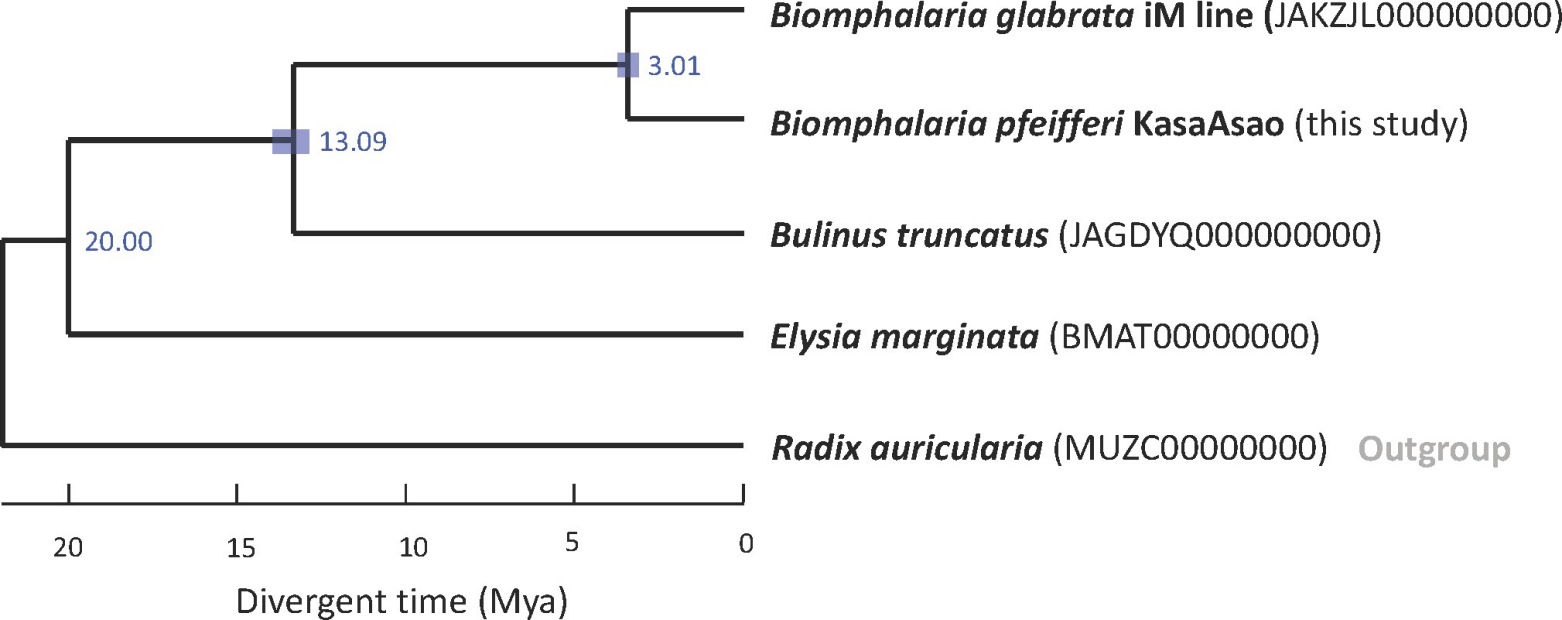
Time tree computed based on species tree with branch length and a constraint point of *Bulinus* emerging 20 Mya, based on the fossil record. The International Nucleotide Sequence Database Collaboration (INSDC) genome IDs on NCBI were listed in the brackets beside each species.

Annotated protein sequences were obtained for *B*. *glabrata* iM line (35,016), *B*. *pfeifferi* (31,894), *Bu*. *truncatus* (26,279), *Elysia marginata* (23,871), and *Radix auricularia* (4,731), and the time tree (Fig 7) was estimated using Orthofinder. When the *Bulinus* emergence time was fixed as the constraint point to 20 Mya based on fossil evidence [71], the split time of *B*. *glabrata* and *B*. *pfeifferi* shown in Fig 7 was determined to be 3.01 Mya (95% CI 2.62–3.47 Mya).

### Comparison of annotated genes among *B*. *pfeifferi*, and three *B*. *glabrata* strains

A total of 12,421 ortho-groups were shared among the 5 genomes sampled in S3 Fig, all of which are representatives of the family Planorbidae, including two genera (*Bulinus* and *Biomphalaria*), and two congeneric species, *B*. *pfeifferi* and *B*. *glabrata*, the latter represented by two isolates originating from Brazil, BB02 and iBS90, and one mixed ancestry isolate, iM line. The category of “shared among all five genomes” far outweighed the number of *Biomphalaria*-specific ortho-groups (n = 1,924). The large number of ortho-groups shared by iM line and iBS90 (n = 2,021) and the relatively large number (n = 1,117) shared by *Biomphalaria* representatives exclusive of *B*. *glabrata* BB02, may reflect differences in sequencing techniques. The number of ortho-groups unique to each of the taxa examined ranged from 164 to 485, not surprisingly with the value for *Bu*. *truncatus* being higher than for the more-closely related *Biomphalaria* representatives.

A total of 7,054 single copy orthologs were detected in common to all five genomes. These comprise the core set of genes among these extant species. These universal single copy genes serve as good bench markers for expected gene content in genomes or transcriptomes. As a reference, the Mollusca Benchmarking Universal Single-Copy Orthologue (BUSCO) single copy orthologs number is 5,295. The higher number of 7,054 we observed among our five genomes is no doubt influenced by their relatively high degree of relatedness as compared to relatedness among all molluscs as a whole.

### Non-coding RNA prediction and annotation summary

We systematically annotated the key types of non-coding RNAs in *B*. *pfeifferi* and were able to obtain 514 tRNAs (with one being a selenocysteinyl tRNA, or tRNA_sec), 757 rRNAs, 77 miRNAs, and 2,954 long non-coding RNAs, or lncRNAs (Table 4). The presence of tRNA_sec in *B*. *pfeifferi* is indicative of its ability to synthesize selenoproteins [111]. Numerous ribosomal RNAs were predicted, likely due to the fact that more repetitive regions were successfully assembled using PacBio accurate long reads.

**Table 4:** Predicted non-coding RNAs in *Biomphalaria* genomes.

ncRNA	Full name	Size (nt)^a^	*B*. *pfeifferi*	*B*. *glabrata* BB02
**tRNA**	transfer RNA	76–90	513	510
tRNA_sec^b^			1	0
Total			514	510
**rRNA**	ribosomal RNA	120–4500		
18S_rRNA			157	
28S_rRNA			146	
5S_rRNA			318	
5_8S_rRNA			136	
Total			757	20
**miRNA**	microRNA	21–23	77	95
**lncRNA**	long non-coding RNAs	>200	2,954	2

Note

^**a**^: Sizes of ncRNAs were collected from Zhang et al. [112]

^**b**^: a type of tRNA dedicated to synthesis of polypeptides containing selenocysteine.

For the miRNAs, 59 of 77 predicted *B*. *pfeifferi* miRNAs show at least 90% identity to *B*. *glabrata* BB02 sequences, leaving 18 miRNAs unique to *B*. *pfeifferi* and 32 unique to *B*. *glabrata*. The shared and unique miRNAs identified in the *B*. *pfeifferi* and *B*. *glabrata* genomes are listed in S6 Table.

### *FReD* genes in the *B*. *pfeifferi* genome

Following the decision tree presented in Fig 2, and criteria for finding FReDs previously established [55], 103 *B*. *pfeifferi* FReDs were identified, including 55 FREPs, 45 sFReDs and 3 FReMs (S7 Table). Most *B*. *pfeifferi* FREPs (49/55) have a signal peptide. Of the 55, 8 had one IgSF loop and 47 had two IgSF domains. The interceding region (ICR) in FREPs refers to the sequence between the IgSF and FBG domains; it forms coiled coils and is usually <150 aa in length. However, we found 21 *B*. *pfeifferi* FREPs with a longer ICR (152~322 aa) and 34 BpFREPs with an ICR of more typical length of <150aa (S7 Table).

Compared with the congener *B*. *glabrata* BB02 and the planorbid *Bu*. *truncatus* with available annotated genomes (Table 5), more FREPs and FReMs were identified in the *B*. *pfeifferi* genome, including two new FReMs (BpFReM2 and BpFReM3, partial). A more detailed overview of *B*. *pfeifferi* FReDs is in S7 Table.

**Table 5:** Summary of *FReDs* in *B*. *pfeifferi* and two related snail genomes.

FReD types	Total # in *B*. *glabrata* BB02 genome [12,55]	Total # in *Bu*. *truncatus* genome [16]	Total # in *B*. *pfeifferi* genome (this study)
**sFReD**	33	129	45
**FReM**	1	0	3
**1-IgSF FREP**	12	0	8
**2-IgSFs FREP**	27	1	47
**Total**	73	130	103

**Fig 8:**
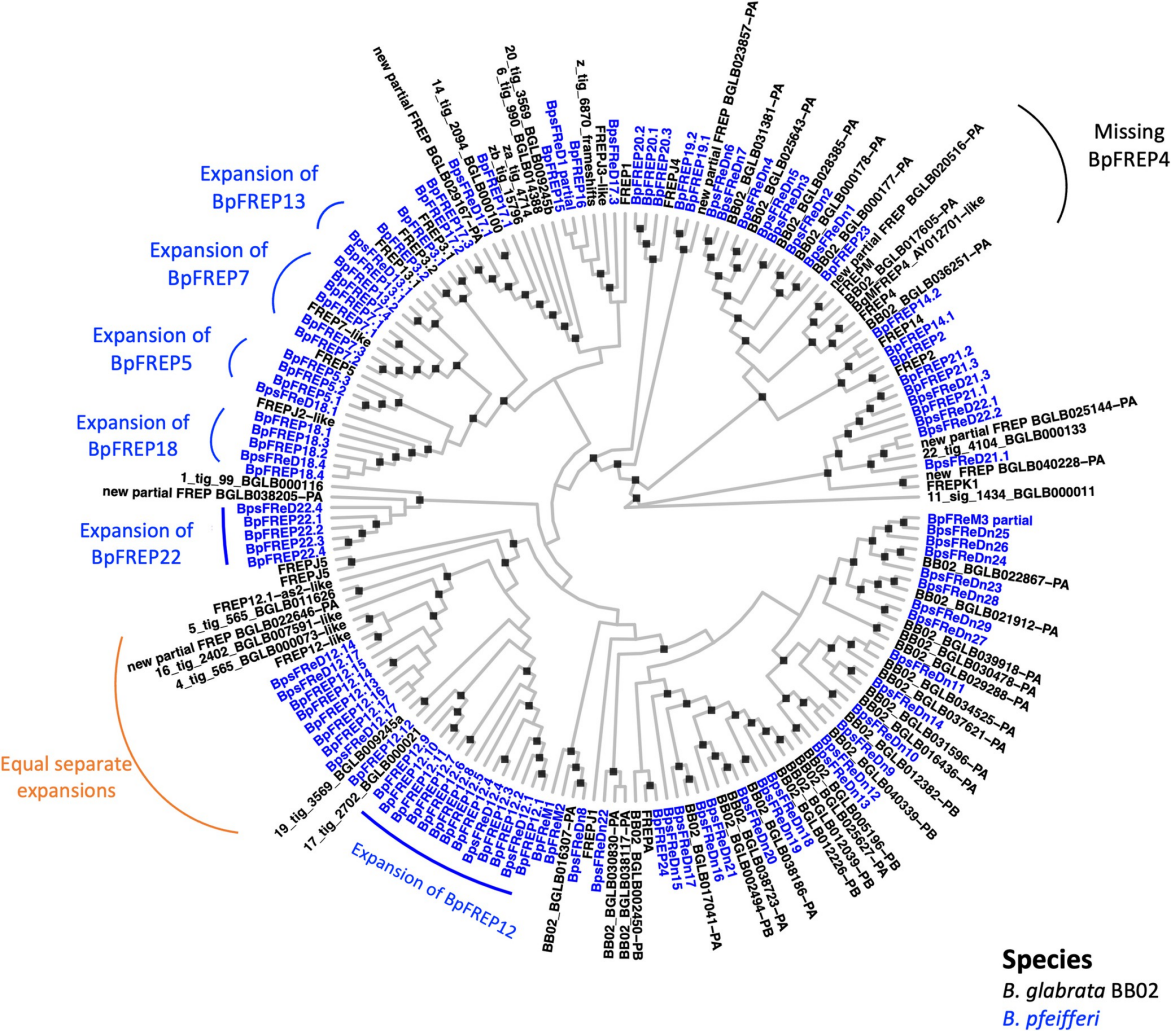
Maximum likelihood tree of FReDs from *B*. *pfeifferi* (blue) and *B*. *glabrata* BB02 (black). The ML tree with 1000 bootstrap replicates was constructed with full length FReDs protein sequences from *B*. *pfeifferi* (this study) and *B*. *glabrata* BB02. Bootstrap values equal or greater than 75% are represented by black squares on the internal nodes. For nomenclature of *B*. *pfeifferi* FReDs see descriptions in this study. Nomenclature of *B*. *glabrata* BB02 FReDs was described in previous studies [12,55,74,75,77,78].

To compare *FReD* genes in the *B*. *glabrata* BB02 and *B*. *pfeifferi* genomes, a Maximum Likelihood tree was generated (Fig 8). Overall, it is striking regarding the extent to which FReDs from the two species are interleaved on the tree, indicative of the close relationship between the South American *B*. *glabrata* and the African *B*. *pfeifferi*. Nonetheless, there are noteworthy divergences between the two species. Note the expansion of BpFREP5, 7, 12, 13, 18 and 22 clusters relative to what is present in *B*. *glabrata*. For example, the 11 members of the BpFREP12 cluster (BpFREP12.1—BpFREP12.11) seem to represent an expansion from a shared ancestral sequence, 17_tig_2702_BGLB000021 being the counterpart in *B*. *glabrata* BB02. Conversely, note the FREP4 cluster in *B*. *glabrata* BB02 that is lacking in *B*. *pfeifferi*.

Because of their responsiveness to schistosome infection shown in previous studies [96], we further examined the sequences of FREP2 and 3 genes from *B*. *glabrata* BB02, BS90 and M line with representatives from the *B*. *pfeifferi* genome, looking for single nucleotide variants (SNV). Similar transcript sequences for FREP2, 3.1, 3.2 were identified (S4 Fig) in the *B*. *pfeifferi* genome (S7 Table). Not surprisingly, for each of these FREPs, the B. *pfeifferi* version was more divergent in sequence than the divergences noted among the representatives obtained from the three *B*. *glabrata* isolates. FREP2, containing a single IgSF domain (see also AlphaFold2 structure predictions below), was relatively conserved between species with SNVs particularly noted in the *B*. *pfeifferi* FBG domain which was also shorter than the corresponding domain in *B*. *glabrata*. Both FREP3.1 and 3.2 contain two IgSF domains (S4 Fig). For FREP3.1, the interceding region (beginning at position 306) was substantially shortened in *B*. *pfeifferi*, and numerous SNVs were identified, once again particularly in the FBG domain, both between strains of *B*. *glabrata* and for *B*. *pfeifferi* in particular. For FREP3.2, overall similarities in sequence were greater between *B*. *pfeifferi* and *B*. *glabrata*, but once again the FBG was somewhat more variable but less so than seen in FREP3.1.

**Fig 9:**
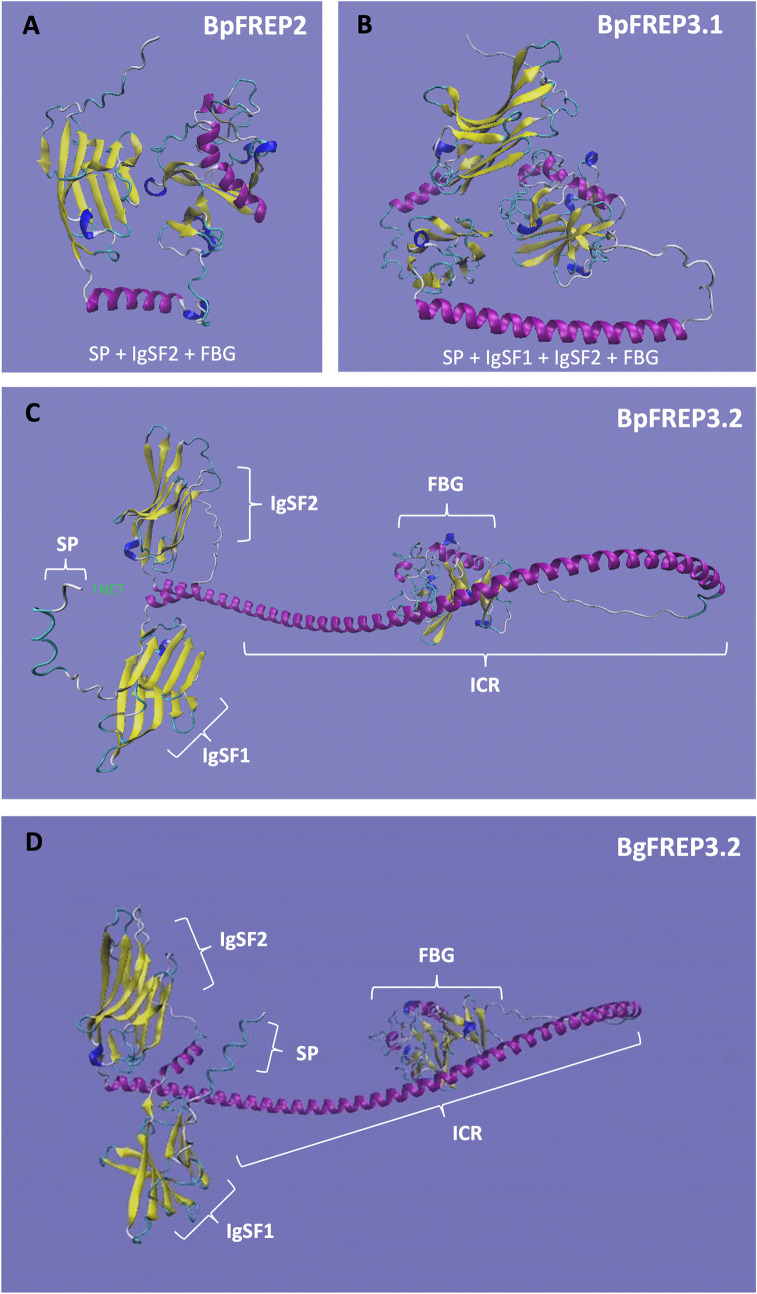
The 3D view of BpFREPs 2, 3.1, 3.2 and BgFREP3.2, predicted by AlphaFold2. Abbreviations: SP, signal peptide; IgSF, immunoglobulin superfamily; ICR, interceding region; FBG, fibrinogen domain. Secondary structures were marked in colored cartoons: alpha helices (3.6 residues per helix turn and 13 atoms ring) in purple, beta sheet in yellow, coils in light grey, beta turn in light blue, and 3(10) helices (3 residues per helix turn and 10 atoms ring) in dark blue.

The 3D structures of BpFREPs were predicted using the AlphaFold2 algorithm and visualized using VMD software (Fig 9). Key functional domains of BpFREPs and the start amino acid (1MET) are indicated in subfigure C. For BpFREP2, note the presence of a single IgSF domain and the relatively short interceding region (alpha helix) that brings the FBG domain close to the IgSF domain. BpFREP3.1 features two IgSF domains but has an interceding region that is truncated in length as compared to other FREPs. For BpFREP3.2, notice the long interceding region more typical of two-IgSF domain containing FREPs. For comparison, Fig 9D shows the AlphaFold2 predicted structure for BgFREP3.2.

### Variable immunoglobulin and lectin domain containing molecules (VIgLs) in the *B*. *pfeifferi* genome

Proteins with lectin domains are often implicated in defense responses in gastropods [80] and their transcription in *B*. *glabrata* has been found to be responsive to exposure to trematodes like *S*. *mansoni* [97]. By BLAST analysis, we found evidence for 63 C-type lectin domain-encoding genes with the length of 53–207 aa in *B*. *pfeifferi* (S8 Table). We focused further attention on two particular categories of lectins, C-type lectin-related proteins (CREP) and galectin-related proteins (GREP) [79].

CREPs have one or two IgSF domains (IgSF1 and IgSF2) and a C-terminal domain CLECT, characteristic of C-type lectins. Based on the gene structures, 20 of the 63 C-type lectin encoding genes contain one or two IgSF loops, thereby fitting the criteria of CREPs (Table 6). Among the 20 CREPs, 11 contain an N-terminal signal peptide and were considered as complete CREPs and 9 were deemed partial CREPs due to missing a signal peptide (S8 Table). One unusual CREP gene (BP27735) contains one IgSF loop, the CLECT domain, and N-terminal repeats with a glutamate-aspartate symporter signature (PR00173). In comparison, only 4 CREPs have been identified in *B*. *glabrata* [79].

**Table 6:** Summary of *B*. *pfeifferi* CREPs.

***B*. *pfeifferi*** **CREPs**	**Immunoglobulin Superfamily (IgSF)**	**IgSF Type**	**with Signal Peptide**	**no Signal Peptide**	**Total**
	1-IgSF loop	IgSF1	5	7	12
		IgSF2	3	2	5
	2-IgSF loops	IgSF1+IgSF2	3	0	3
	**Total**		11	9	20

The 43 remaining C-type lectin domain-encoding genes either contain the CLECT domain only or the CLECT domain in combination with some other conserved motif, such as an Apple-like domain, Rhodopsin 7-helix transmembrane protein, or Trimeric LpxA-like superfamily member (S8 Table).

With respect to GREPs which are comprised of an N-terminal signal peptide, one or two IgSF domains, IgSF1 and IgSF2, and a C-terminal galectin domain GLECT, none were found in *B*. *pfeifferi* as compared to one known from *B*. *glabrata* [79]. However, a gene (BP09483) containing an N-terminal signal peptide and a galectin/galactose-binding lectin (cd00070) was identified.

We explored the location within the whole genome of the VIgLs gene families in the *B*. *pfeifferi* genome (S5 Fig). The genes encoding FREPs (IgSF + FBG), free-standing IgSFs, sFReDs, CREPs (IgSF + C-type lectin) and GREPs (IgSF + galectin) were identified in different scaffolds in the *B*. *pfeifferi* genome (in S5 Fig).

With the exception of a few scattered members, FREPs in *B*. *pfeifferi* were found clustered within hotspots on two scaffolds (BP014 and BP029), both of which reside tin linkage group LG13. These two scaffolds are 8.2 Mb apart, separated by scaffolds BP031 and BP062. No obvious clustered hot spots were found for other VIgLs like CREPs, or isolated IgSF or sFReDs.

### AIG genes in the *B*. *pfeifferi* genome

The AIG (avrRpt2-induced gene) family are characterized by the presence of an AIG1 domain. Members of the AIG gene family are frequently represented as GIMAPs (GTPase of the immunity associated protein family), characterized by presence of the AIG1 domain along with coiled-coil domains. AIG family members first attracted attention in *B*. *glabrata* because some were found to be constitutively highly expressed in snails resistant to *S*. *mansoni* as compared to schistosome-susceptible snails [113]. An expanded AIG gene family with 91 members (64 GIMAPs and 27 AIG genes without coiled-coils) was discovered in the *B*. *glabrata* genome [81].

In the *B*. *pfeifferi* genome, 69 AIG genes were identified (S9 Table), of which 62 have sequence similarity in the *B*. *glabrata* BB02 genome (47 GIMAPs and 15 AIGs). The remaining 7 which are distinctive to *B*. *pfeifferi* include 2 GIMAPs and 5 AIGs. As is the case with *B*. *glabrata*, most of the *B*. *pfeifferi* AIGs contain only 1 AIG1 domain, but a few contain two AIG1 domains, or a special domain, such as a Death domain, or a Hedgehog/Intein domain. More details can be found in S9 Table.

### Sequence similarity searches in the *B*. *pfeifferi* genome for schistosome-resistance complexes identified from *B*. *glabrata* genome-wide association studies (GWAS)

Several *B*. *glabrata* GWAS studies published prior to 2022 [103–106] predicted candidate genes in particular chromosome regions associated with resistance to *S*. *mansoni*. Using the list of potential candidate resistance genes summarized from these studies by Lu et al., (2022) [97] and shown in S4 Table, similarity searches were undertaken in the *B*. *pfeifferi* genome with the query sequence coverage and identity ≥85% as cutoff values. We also searched the recently published GWAS study of *B*. *glabrata* iM line and iBS90 genomes by Bu et al., 2022 [40]. Results are detailed in S10 and S11 Tables.

The general synteny and composition of genes within the *B*. *glabrata* GWAS resistance regions can be confirmed for *B*. *pfeifferi*. For instance, three *B*. *pfeifferi* fructose-bisphosphate aldolase genes similar in sequence to genes (Bg26681, Bg24773, and Bg23696) of the Guadeloupe Resistance Complex [114] were identified though their exact location and size differ. Similarly, multiple histone H4 genes [114], multiple palmitoyltransferase [104], and zinc finger protein-encoding genes [104] were identified and located in different scaffolds of the *B*. *pfeifferi* genome (S10 Table). In reference to the resistance loci from iM line/iBS90 genomes [40], multiple genes with similar sequences were again verified to be present in the *B*. *pfeifferi* genome, for instance: 39S ribosomal proteins, DNA ligase 1, kinesin-like proteins, potassium voltage-gated channel protein Shaw-like, retinol dehydrogenase proteins, thioredoxin domain-containing proteins 12-like isoform X2, transcription elongation factors, transcription factors, transient receptor potential cation channel subfamily A member 1-like isoform X1, and transmembrane proteins (S11 Table). More detailed analyses will be required to determine if and how the sequences of individual genes within the resistance complexes may vary from *B*. *glabrata* to *B*. *pfeifferi* (see discussion), and the functional roles genes identified by GWAS studies might actually play in schistosome resistance.

### Biomphalysins in the *B*. *pfeifferi* genome

Biomphalysins are proteins averaging 575 residues in length containing an N-terminal signal peptide for secretion (17–23 amino acids), an aerolysin domain consisting of 5 β-strands with an insertion loop corresponding to the pore-forming transmembrane domain (TMD) [82]. Twenty-three biomphalysins have been reported from *B*. *glabrata* and are of particular interest because they represent toxin genes acquired by horizontal transfer from bacteria, and they have been shown to be toxic to schistosome sporocysts so have potential relevance in immune defense [82,83].

Using criteria provided for *B*. *glabrata* biomphalysins, the transmembrane domains were searched using the PRED-TMBB server (PREDiction of TransMembrane Beta Barrels proteins) [88,89], and 14 putative biomphalysins were identified in the *B*. *pfeifferi* genome (S12 Table), 8 of which had a predicted signal peptide for secretion. Six *B*. *pfeifferi* biomphalysins were considered as complete using the criteria by Pinaud et al. (2021) [82]. Interestingly, the transcript BP08351-RA (coding gene ID: BP08351) contained a signal peptide and sequence of 178aa which lacks intact aerolysin or pore-forming domains. However, it did match 178aa of *B*. *glabrata* biomphalysin 10 (partial, QUJ23862.1, 575aa) with 97.8% of identity and 100% coverage. It’s possible either a downstream aerolysin domain was missed during genome assembly, or a deletion occurred within this gene. Therefore, BP08351 is included in the *B*. *pfeifferi* genome as a partial biomphalysin (S12 Table).

### Heat shock protein 70 (HSP70) in the *B*. *pfeifferi* genome

Heat shock proteins (HSPs) are known to play a vital role in the synthesis, transport and folding of proteins. HSP70s are unique in the HSP family due to their high degree of conservation [115]. They have been repeatedly identified in molluscs and in *B*. *glabrata* (11–21 HSPs) [116–118], and have been implicated in *B*. *glabrata* defense reactions [119,120]. Several (41) HSP70s were found in the *B*. *straminea* genome [13].

Eleven HSP70s with the conserved HSPA domain (cd10233, or PTHR19375) were identified in the *B*. *pfeifferi* genome (S13 Table), four of which (BP17603, BP17604, BP17605 and BP17606) were in tandem. Considering their similar overall structures and areas of large sequence overlap, three of these genes (BP17603, BP17604 and BP17605) may have a common originating sequence.

### G-protein coupled receptors (GPCR) and Toll-like receptors (TLR) in the *B*. *pfeifferi* genome

G-protein coupled receptors (GPCR) play an important role in environmental sensing [48,121] and 241 seven transmembrane domain GPCR-like genes belonging to 14 subfamilies and clusters were found in the *B*. *glabrata* BB02 genome [12]. We have previously noted remarkably different patterns of GPCR expression in *B*. *glabrata* snails susceptible or resistant to *S*. *mansoni* infection [97]. In this study, we applied massive BLAST analysis of the *B*. *pfeifferi* genome to NCBI databases, combined with InterProScan and Uniport for GPCR domains prediction, and identified 219 putative GPCRs (S14 Table). There were 16 different types of GPCR domains identified within the GPCR family by domain prediction and NCBI annotation, four of which were prominently represented among *B*. *pfeifferi* GPCRs. For instance: 96 GPCRs contained the domain of “Rhodopsin 7-helix transmembrane proteins” (G3DSA:1.20.1070.10); 39 GPCRs had a “Rhodopsin-like GPCR superfamily signature” (PR00237); 31 GPCRs contained “G_PROTEIN_RECEP_F2_4 DOMAIN-CONTAINING PROTEIN” (PTHR45902), and 10 GPCRs contained “MYOSUPPRESSIN RECEPTOR 1, ISOFORM B-RELATED” (PTHR46273). Considering the number and complexity of GPCRs revealed in *B*. *glabrata* and *B*. *pfeifferi*, and their responsiveness to schistosome infection, the GPCRs would seem to comprise a fruitful subject for future investigation.

Toll-like receptors (TLRs) are known to be important pathogen recognition receptors (PRRs) responding to pathogen-associated molecular pattern molecules or PAMPs, triggering activation of immune signaling pathways [12,122–124]. In the *B*. *glabrata* BB02 genome, 27 complete, 9 partial, and 20 TLR pseudogenes were identified [12]. TLRs have been implicated in the response of *B*. *glabrata* to *S*. *mansoni* infections [84,125]. In *B*. *pfeifferi*, by BLAST analysis, 43 TLRs were identified. Of these 43, 17 are considered as complete TLRs with an N-terminal signal peptide, a series of leucine-rich repeats (LRRs), a transmembrane domain, and an intracellular Toll/Interleukin-1 receptor (TIR) domain. The remainder are categorized as TLR partials because they lack one or more of the conserved domains (S15 Table).

### Genes possibly involved in sexual attraction in the *B*. *pfeifferi* genome

Secreted protein pheromones involved in sex recognition, aggregation, spawning, and larval metamorphosis are known from molluscs [86,126–130], including the *Aplysia californica/brasiliana* pheromonal complex of attractin, seductin, enticin, and temptin. Similar to previous observations for *B*. *glabrata* [12], we found evidence for the presence of only temptin in *B*. *pfeifferi* [27]. As in *B*. *glabrata* BB02 and iBS90 genomes, we found 12 predicted partial temptin-like genes in *B*. *pfeifferi* (that did not contain a signal peptide), whereas *B*. *glabrata* iM line had 11 (S6 Fig and S16 Table). Additionally, one temptin-like gene was found in *B*. *pfeifferi*, that contained a signal peptide (S16 Table). All 13 *B*. *pfeifferi* genes contained a conserved Ca^2+^ binding EGF-like domain (DSDXD). All 13 predicted temptin-like (including ones without signal peptides) genes also grouped closely with *B*. *glabrata* temptin-like gene, including Bp30710RA with XP_013086708.1 from *B*. *glabrata*, a recombinant form of which was shown to attract *B*. *glabrata* in T-maze experiments (S6 Fig). Considering the preference of *B*. *pfeifferi* for selfing relative to outcrossing for *B*. *glabrata*, we considered there might be notable differences in their temptin genes, but did not find this to be the case. More T-maze experiments might reveal functional differences among temptins from the two species not obvious from their sequences.

We found two hits (gene IDs BP16290 and BP22349) to *Aplysia* attractin, XP_012937027.2. However, the inferred proteins are much larger than *Aplysia* attractin proteins which are < 100 aa in length, and do not have key conserved attractin residues. It is likely that these *B*. *pfeifferi* proteins have other functions not related to sexual attraction or aggregation.

## Discussion

The *B*. *pfeifferi* genome sequence greatly increases our knowledge base for this understudied species, one that plays a pivotal role in maintaining schistosome transmission across much of the Afrotropical region and in southwest Asia. *B*. *pfeifferi* is well-known for its widespread involvement in *S*. *mansoni* transmission, and in broad terms should be considered a highly relevant vector species representing remarkable compatibility with schistosome infection.

The genome sequence was obtained using PacBio high-fidelity (HiFi) sequencing technology which generates accurate long reads (up to median 25Kb, accuracy exceeding 99.9% or >Q30) [131]. HiFi sequencing provided the resolution needed to separate high similarity repeats and alleles across large genomic regions [131]. This enabled us, for example, to identify additional rRNAs in the genome.

Surprisingly, the *B*. *pfeifferi* genome is smaller (772 Mb) than known genomes for other *Biomphalaria* or *Bulinus* species, and it contains proportionately about the same amount of repetitive DNA as genomes of close relatives (S2 Table and S2 Fig). The number of gene models (31,894) is comparable to other known members of its gastropod family Planorbidae (range of 26,729 to 43,340). Due to the limitation of current sequencing technology, repeats (especially long repeats) can collapse during the *de novo* genome assembly process, thereby resulting in a smaller assembled genome size than expected. However, in this study, the reduction of genome size in *B*. *pfeifferi* could not be explained only by assembly challenge from repeats or limitation of sequencing technology. As results above have shown, two independent non-assembly-based genome size estimation approaches (K-mer method and fluorescence methods) returned consistent results that the *B*. *pfeifferi* genome size is smaller than the *B*. *glabrata* genome. Additionally, the PacBio HiFi reads in this study have a mean length of 18Kbp and 37x depth, which leaves less opportunity for collapsing repeats. Therefore, despite the caveat that the *B*. *pfeifferi* genome might have a reduced genome size due to repeats collapsing during the assembly process, we have reached the conclusion that the *B*. *pfeifferi* genome size is just naturally smaller than the *B*. *glabrata* genome.

Genome-based k-mer analysis (Fig 3) suggested a relatively low degree of heterozygosity in *B*. *pfeifferi* as compared to other planorbid genomes. This is also supported by heterozygosity of SNP genotypes from RNA-Seq data: for *B*. *pfeifferi*, this ranged from 7–28%, whereas for outbred *B*. *glabrata* M line the value was 39%. The values for *B*. *pfeifferi* were closer to the 6% value noted for *B*. *glabrata* iM line which had been deliberately inbred for 81 generations. The low degree of heterozygosity for *B*. *pfeifferi* is consistent with the strong, but not exclusive, predilections for self-crossing as opposed to out-crossing for this snail species [132], a trait not shared by *B*. *glabrata* which is naturally a preferential out-crosser [133]. In nematodes and plants, there is a trend for selfing species to have smaller genomes than closely-related out-crossing representatives, with one explanation being the loss of genes associated with mating [134]. So, for *B*. *pfeifferi*, even though it is not an exclusive selfer, both diminished heterozygosity and smaller genome sizes may be consequences of its relative reliance on selfing. Sequencing of additional individuals of *B*. *pfeifferi*, and of different schistosome vectors also known to be selfers (e.g. *Bulinus forskalii* relative to its out-crossing congeners) [1], will be of interest in revealing the extent to which representative of different selfing lineages share the trait of limited heterozygosity, and the extent to which they differ in their genome size.

Building on the pioneering work of the genome project for field-collected *B*. *glabrata* BB02 [12] and of subsequent PacBio long reads sequencing projects for commonly used *B*. *glabrata* M line and BS90 lab strains [40], 18 well-supported linkage groups have been inferred for *B*. *pfeifferi*, matching the known haploid number of 18 chromosomes for this and other species of *Biomphalaria*. A total of 53.5% of the *B*. *pfeifferi* genome exhibits synteny with the *B*. *glabrata* iM line genome assembly [40], as might be expected given the hypothesized origin of *B*. *pfeifferi* and the remaining 11 species of African *Biomphalaria* from the trans-Atlantic colonization of a *B*. *glabrata*-like ancestral snail [11]. Based on rates of sequence divergence among available genomes for relevant gastropod species, we estimated a divergence time between *B*. *glabrata* and *B*. *pfeifferi* of 3.01 million years, a rate that falls into the range of 2.62–3.47 million years proposed earlier as the separation time for these two species [2]. Our estimate is based on the oldest known presence of *Bulinus* in the fossil record which does not necessarily reflect the *earliest* appearance of this genus in evolutionary history. We return to a discussion of the implications of this study with respect to relationships between *Biomphalaria* species and schistosomes and other trematodes below.

As compared to other available schistosome vector snail genomes, we provide a relatively comprehensive list of the non-coding RNA genes found, including rRNAs, tRNAs, miRNAs, and lncRNAs. In the *B*. *glabrata* BB02 genome, two lncRNAs were predicted [12] as compared to 2,954 lncRNAs predicted for *B*. *pfeifferi*. As our knowledge of genome regulation improves, the significance of the many lncRNAs identified will become more apparent: they will provide insights into regulation of gene expression at multiple levels (epigenetic, transcriptional and post-transcriptional).

Among the many gene families identifiable in *Biomphalaria* genomes [12], we chose to focus attention on those that were most familiar to us, and that have been implicated in previous studies for a role in immune defense. Prominent among these is the FReD family. In general, *B*. *glabrata* and *B*. *pfeifferi* had similar overall FReD profiles, with comparable numbers of sFReDs, FReMs and FREPs. By contrast, *Bu*. *truncatus* was found to have only a single FREP and relatively more sFReDs. The number of FREPs found in *B*. *pfeifferi* (55) exceeded that found in *B*. *glabrata* (39). The sole FREP from *Bu*. *truncatus* possesses two IgSF domains which are relatively dissimilar from the IgSF domains found in *B*. *pfeifferi* or *B*. *glabrata* BB02/iM line/iBS90. In fact, IgSF domains in *Bu*. *truncatus* FReDs were only retrieved from predicted 3D protein structures showing the typical 8-beta sheets featured in IgSF domains. It will be of particular interest to chart the expansion or contraction of FReDs across the Planorbidae as more data become available. *Bulinus* is a relatively early diverging genus in the family and is even placed by some in a separate family, the Bulinidae. In contrast, *Biomphalaria* represents a more derived lineage [135,136]. Provision of sequence data for species representing other major planorbid lineages will be revealing in this regard.

The three-dimensional structures predicted by Alphafold2 for *B*. *pfeifferi* FREPs were striking, notable both for their differences from Alphafold2 predictions for *Bu*. *truncatus* FREPs, and for how impactful the interceding region is in dictating their overall structure. With respect to four FREPs noteworthy in *B*. *glabrata* for their responsiveness to *S*. *mansoni* infection [55], FREPs 2, 3.1 and 3.2 and 4, we found the first three to have identifiable sequence similarity in *B*. *pfeifferi*. Generally, the versions from *B*. *pfeifferi* were more divergent in base composition than were the various *B*. *glabrata* representatives. It was somewhat surprising that the fibrinogen domains appeared more prone to variation than the IgSF domains. Further analysis of the functional roles of FREP IgSF and fibrinogen domains will be facilitated by use of 3-dimensional structures such as those shown in Fig 9, enabling their roles in available models of interaction with *S*. *mansoni* [137] to be more thoroughly defined. This may in turn lead to rational development of schistosome resistance factors that might be engineered for expression in transgenic snails [138].

It was noteworthy that no sequence similar to FREP4 was found in *B*. *pfeifferi*. This gene was found to be down-regulated early during the course of *S*. *mansoni* infection in schistosome-susceptible *B*. *glabrata* M line snails, but was persistently overexpressed in *S*. *mansoni*-resistant BS90 snails following exposure to infection [97]. This may reflect that susceptibility to *S*. *mansoni*, if linked to FREP4, might result from two different processes: down-regulation in susceptible strains of *B*. *glabrata*, or loss of the gene in *B*. *pfeifferi*. In contrast, FREP2 and 3 have the same copy numbers in the two related *Biomphalaria* species, suggesting their stable maintenance is significant. Furthermore, not only has FREP4 disappeared in *B*. *pfeifferi* but a novel FREP, FREP12, is present, indicative of the dynamic nature of the FReD gene family, exhibiting both loss and expansion.

With respect to other molecules with IgSF domains, CREPs have both an IgSF domain and a C-terminal C-type lectin domain [79]. More CREPs were found in *B*. *pfeifferi* (20) than in *B*. *glabrata* (4). Their role in schistosome interactions requires more study, but proteins with sequence similarity to CREP IgSF sequences have been found adherent to *S*. *mansoni* sporocysts [12]. Previous transcriptome studies comparing the responses of *B*. *glabrata* susceptible or resistant to *S*. *mansoni* have also found distinctive response profiles for C-type lectins which normally lack an IgSF domain [97]. The number of C-type lectins has not been determined for *B*. *glabrata* although 111 lectins representing multiple categories are known to be present [97]. *B*. *pfeifferi* has 43 C-type lectins and *Bu*. *truncatus* has 101 mostly C-type lectins [16]. Further comparative study is needed to learn if consistent patterns in lectin transcription occur in parasite-exposed snails, particularly in snails actively shedding schistosome cercariae, as was noted by Lu et al. [97].

Another gene family known from *Biomphalaria* is the AIG gene family, which is expanded in *B*. *glabrata* with 91 members, with some members known to be expressed at constitutively high levels in snails resistant to *S*. *mansoni* [81]. *B*. *pfeifferi* also has an expanded AIG family containing 69 members. The presence and size of this gene family in other planorbid snails is as yet unknown, as is the role they might play when up-regulated in schistosome-resistant snails.

*B*. *glabrata* has 23 pore-forming-encoding biomphalysin genes [82], and *B*. *pfeifferi* was found to have 14 representatives, strongly suggesting these horizontally acquired genes of bacterial origin have survival value for *Biomphalaria*. Biomphalysins have been shown to adhere to and damage schistosome larvae [139,140]. Toll-like receptors (TLRs) are believed to play a key role in defense of invertebrates from pathogens by serving as sensors that can activate signaling pathways leading to the production of immune effectors. A role for TLRs in defense against *S*. *mansoni* sporocysts has been noted by [84]. In *B*. *pfeifferi*, 43 complete, partial or pseudogene TLRs were recovered, whereas *B*. *glabrata* was found to have 56, and *Bu*. *truncatus* 123.

Another prominent group of molecules implicated in *Biomphalaria* defense from schistosomes are HSP70 genes [12,120]. The invasive schistosome vector species *B*. *straminea* has an enriched complement of HSP70s (41) relative to *B*. *glabrata* (11–21) and *B*. *pfeifferi* with 11. In molluscs, particularly bivalves, HSP70s are among the most frequently investigated molecules and are believed to play an important role in protection from thermal stress [141]. It will be of interest to learn if increased complements of HSP70 genes facilitate the invasiveness shown by *B*. *straminea* or other invasive gastropods. The role of several of these gene families in snail-schistosome interactions remains to be more fully investigated, and the perspective offered by comparative genomics will prove to be an insightful way forward.

One of the most distinctive features of the biology of *B*. *pfeifferi* is its strong preference for self-fertilization, often viewed as an advantageous way to quickly re-populate fluctuating habitats [142]. One possible factor influencing this behavior is the extent to which they produce proteins known to influence aggregation or mating behavior. Four different categories of proteins have been shown to affect the aggregation and mating behavior of *Aplysia*: temptin, attractin, seductin, and enticin [85,128,143]. Only temptin is known to be produced by *B*. *glabrata* [12,144]. We found 12 partial temptin-like genes (and 1 that contained a signal peptide) in *B*. *pfeifferi*, comparable in number to the outcrossing *B*. *glabrata* with 12 temptin genes.

In the *B*. *glabrata* transcriptomic study of Lu et al. [97], five temptins were overexpressed in unexposed schistosome-resistant snails as compared to unexposed susceptible snails. It was also noticed that for susceptible snails shedding *S*. *mansoni* cercariae, four temptin genes were up-regulated while one was down-regulated relative to unexposed controls. Why snails effectively castrated by *S*. *mansoni* would be producing more temptins is somewhat baffling if their role is only in attraction of conspecifics. In *B*. *pfeifferi* exposed to *S*. *mansoni* [27], some temptin-like genes were highly expressed early in the course of infection and then down-regulated once the snails began shedding cercariae, a pattern that seems more compatible with what might be expected from a castrated snail. Transcription of some *B*. *pfeifferi* temptin-like genes was unaffected by *S*. *mansoni* infection, suggesting they might have other functions unrelated to reproduction, a supposition supported by other studies [87,145,146], including a role in biomineralization in freshwater mussels [89]. Further studies are needed to determine the function of each of the *B*. *pfeifferi* temptin genes and how they differ from other *Biomphalaria* species. Differences in mating behavior between species might be influenced by expression of distinctive suites of temptins, or by sequence differences among the encoded proteins, hypotheses deserving additional study.

Another group of molecules with a largely unappreciated impact on the biology of freshwater gastropods are the membrane-associated signal-receiving G-protein coupled receptors (GPCRs). Not surprisingly, several GPCRs are present in *B*. *pfeifferi* (219), though fewer than reported from *B*. *glabrata* (241) or *Bu*. *truncatus* (709). Further specific analysis of the complements of GPCRs present in the different schistosome vector snails is clearly a topic deserving additional study, not only with respect to how they respond to the presence of schistosome parasites *per se*, but also how it might relate to differences in their ability to perceive a huge range of environmental cues. Transcriptomic results [97] highlight how responsive GPCRs seem to be upon exposure to schistosome infections, both in snails susceptible or resistant to infection.

The schistosome-resistance gene complexes identified by genome-wide association studies in *B*. *glabrata* [40,103–106] offer another relevant point of comparison with *B*. *pfeifferi*. Not surprisingly, comparable regions with similar gene composition were found in the *B*. *pfeifferi* genome. Nonetheless, both *B*. *glabrata* and *B*. *pfeifferi* are comprised of individuals that largely, but not necessarily exclusively, exhibit a natural state of susceptibility to *S*. *mansoni* across their respective geographic ranges [147]. So, under natural circumstances it seems resistance to this parasite has not been strongly selected for. It is also pertinent that *S*. *mansoni* is just one of several species of castrating digenetic trematodes known to infect either snail species: both support diverse communities of trematodes [25,148–150]. Whether this results in more generic responses to minimize the impact of all trematodes, or produces more tailored trematode-species specific responses is a topic that requires further study. For some species like *B*. *pfeifferi* that occupy habitats subject to rapid change, the threat imposed by parasites and the presumed benefits offered by regular out-crossing may be offset by the advantages of rapid colonization achieved by selfing, especially if the risk of trematode infection is small.

Lastly, consider the peculiar colonization history of a *B*. *glabrata*-like ancestor from South America to Africa in an interval, as again suggested by this study, probably less than five million years ago. Some caution is required in interpreting this date because, although it is agreement with other estimates of the divergence’s times of the two species [2], our present calibration used as an anchor point a 19–20 Mya divergence time for *Bulinus*, and this may underestimate the actual origin time of demonstrable *Bulinus* snails. Similar issues have been reported by others in attempts to estimate divergence times [69,70]. It would be the case that the descendants that subsequently diversified in Africa, with *B*. *pfeifferi* being the first-diverging African *Biomphalaria* species among them [2,11], would have benefitted by their colonist ancestor’s experience to trematode exposure in South America, but that exposure history would not have included exposure to mammalian schistosomes like *Schistosoma*, which were Afro-Asian in origin and distribution. A snail species likely to have encountered the progenitor of *S*. *mansoni* or the related *S*. *rodhaini* in Africa is *B*. *pfeifferi*. The susceptibility shown by *B*. *pfeifferi* to *S*. *mansoni* today may reflect this early period of intimate association. Interestingly, any specific accommodation *B*. *pfeifferi* and related African snails may have made to *S*. *mansoni* and its relatives would be independent from whatever specific accommodation *B*. *glabrata* may have made with *S*. *mansoni* (they may have shared a general accommodation strategy to trematode given their relatedness, however). This would be because *S*. *mansoni* did not encounter *B*. *glabrata* until 400–500 years ago, when *S*. *mansoni*-infected people were first transported from Africa to the New World. It would prove interesting to learn if the accommodations made (if any) by *B*. *pfeifferi* to *S*. *mansoni* prove to have any resemblance to those made independently by *B*. *glabrata* to *S*. *mansoni* at a later time. In other words, how prominent might schistosome resistance genes be in either species in nature, and to what extent might selection have converged on the same genes in each species as they independently encountered *S*. *mansoni*? Also, resistance may be manifested very differently between the two species, being present in distinct largely self-propagating lineages in *B*. *pfeifferi*, whereas resistance traits might segregate more freely among out-breeding *B*. *glabrata*. Once again, comparative genomics as offered by this and other forthcoming genome studies, as for species like the African out-crossing species *Biomphalaria sudanica*, will help us delve into these and related subjects in depth.

## Supporting information

S1 Table Summary of complete and fragmented BUSCOs in the four snail genomes. (XLSX)

S2 Table Repeat elements in the *B*. *pfeifferi*, *B*. *glabrata* iM line and iBS90 genomes. Notes: 1: * Most repeats fragmented by insertions or deletions have been counted as one element. 2: Repeats were identified using RepeatMasker 4.0, with customized repeat libraries, which was generated using RepeatModeler 2.0.1 followed by filtering out protein like sequences using BLAST and InterProScan domain prediction (details in Method).
(XLSX)

S3 Table *B*. *pfeifferi* contigs assigned to 18 inferred linkage groups based on comparison to *B*. *glabrata* iM line. (XLSX)

S4 Table Summary of structural variation (> 1Kb) for *B*. *pfeifferi* in comparison to *B*. *glabrata* iM line. (XLSX)

S5 Table Annotations and sequence similarities for genes within the inverted region on LG9 of *B*. *pfeifferi* genome in comparison to *B*. *glabrata* iM line. Multiple sheets in the Excel table recorded the details of the inversion between the two genomes: provided are inversion region locations; annotations and sequence similarities of 24 reciprocal best hits; annotations of all genes within the inversion region in the two genomes; ordered gene locations and MCscan scores for genes within the inversion region.
(XLSX)

S6 Table Predicted miRNAs in the *B*. *pfeifferi* and *B*. *glabrata* BB02 genomes. (XLSX)

S7 Table Annotated features for *FReD*s identified within the *B*. *pfeifferi* genome. (XLSX)

S8 Table C-type lectin related proteins (CREPs) identified within the *B*. *pfeifferi* genome. (XLSX)

S9 Table AIG/GIMAP gene family identified within the *B*. *pfeifferi* genome. (XLSX)

S10 Table Summary of sequence similarity to genes identified in the genome-wide associated studies (GWAS) published prior to 2022 in the *B*. *glabrata* genome. (XLSX)

S11 Table Summary of sequence similarity to genes identified in the recent published GWAS study by Bu et al., (2022) [40] of *B*. *glabrata* iM line and iBS90 genomes in the *B*. *pfeifferi* genome. (XLSX)

S12 Table Summary of biomphalysins in the *B*. *pfeifferi* genome. (XLSX)

S13 Table Summary of heat shock protein 70s (Hsp70s) in the *B*. *pfeifferi* genome. (XLSX)

S14 Table Summary of G-protein coupled receptors (GPCRs) in the *B*. *pfeifferi* genome. (XLSX)

S15 Table Summary of Toll-like receptors (TLRs) in the *B*. *pfeifferi* genome. (XLSX)

S16 Table Summary of temptin-like genes in the *B*. *pfeifferi* genome. Note: Predicted genes were run in BLAST and then searched for signal peptides and the conserved Ca^2+^ predictive binding site, DSDXD. Only one of the temptin-like genes contained a signal peptide.
(XLSX)

S1 Fig RNA-Seq SNP genotypes from individual snails showing high proportions of identified homozygous SNPs. The bar shows the proportion of homozygous (blue), or heterozygous (orange) SNPs found in the individual snails used in RNA-Seq studies of *B*. *glabrata* [97] and *B*. *pfeifferi* [27,28,48]. Abbreviations: Bg, *B*. *glabrata*; Bp, *B*. *pfeifferi*; Sm, *S*. *mansoni*; dpe, days post-exposure; Moll, molluscicide treated; R#, replicate number. BpSmMollR3 means *B*. *pfeifferi* infected with *S*. *mansoni* and treated with molluscicide, the 3^rd^ replicate.
(TIF)

S2 Fig Comparisons of representation (in millions of base pairs) of annotated repetitive elements in *B*. *pfeifferi* and two *B*. *glabrata* genomes, iM line and iBS90. Abbreviations: Bg, *B*. *glabrata*; Bp, *B*. *pfeifferi*
(TIF)

S3 Fig Upset plot showing ortholog groups shared among five snail species with annotated genome. (TIF)

S4 Fig SNV analysis of FREP2 and 3 among *B*. *pfeifferi* and *B*. *glabrata* BB02, M line and BS90. (TIF)

S5 Fig Genomic distribution of key VIgLs gene families in *B*. *pfeifferi* genome. The free-standing IgSF domain-containing genes (free-standing IgSF in purple) and variable immunoglobulin and lectin domain-containing molecules (VIgLs) gene families including FREPs (blue), CREPs (yellow), and sFReDs (green) were marked on scaffolds according to predicted genomic locations.
(TIF)

S6 Fig Maximum Likelihood tree of temptin-like genes from this study and from other *Biomphalaria* temptin and temptin-like genes from GenBank. The evolutionary history was inferred by using the Maximum Likelihood method and JTT matrix-based model from Jones et al. (1992) [151]. An (*) indicates bootstrap values greater than 90%. All 13 *B*. *pfeifferi* temptin-like (including those without signal peptides) genes are shown in bold and each clade is color coded. All the *B*. *pfeifferi* temptin-like genes group with *B*. *glabrata* temptin-like genes, either from *B*. *glabrata* BB02, iM line, or iBS90 genomes. One temptin-like gene grouped with BB02, two grouped with iM line, one grouped with iBS90, and the rest of the 8 temptin-like genes all grouped together. The clade with the star is the *Bg*Tempin clade from Pila et al. (2017) [144], a study which demonstrated *B*. *glabrata* is attracted to this protein. The tree with the highest log likelihood (-1627.03) is shown. A discrete Gamma distribution was used to model evolutionary rate differences among sites (5 categories (+G, parameter = 2.4126)). The rate variation model allowed for some sites to be evolutionarily invariable ([+I], 1.09% sites). The tree is drawn to scale, with branch lengths measured in the number of substitutions per site. This analysis involved 52 amino acid sequences. All positions containing gaps and missing data were eliminated (complete deletion option). There was a total of 46 positions in the final dataset. Evolutionary analyses were conducted in MEGA X by Kumar et al., (2018) [71].
(TIF)
