# Supplementary figures and images for: A genome sequence for Biomphalaria pfeifferi, the major vector snail for the human-infecting parasite Schistosoma mansoni
Source: PLoS Negl Trop Dis. 2023 Mar 24;17(3):e0011208. doi: 10.1371/journal.pntd.0011208 (PMC10075465; doi:10.1371/journal.pntd.0011208)

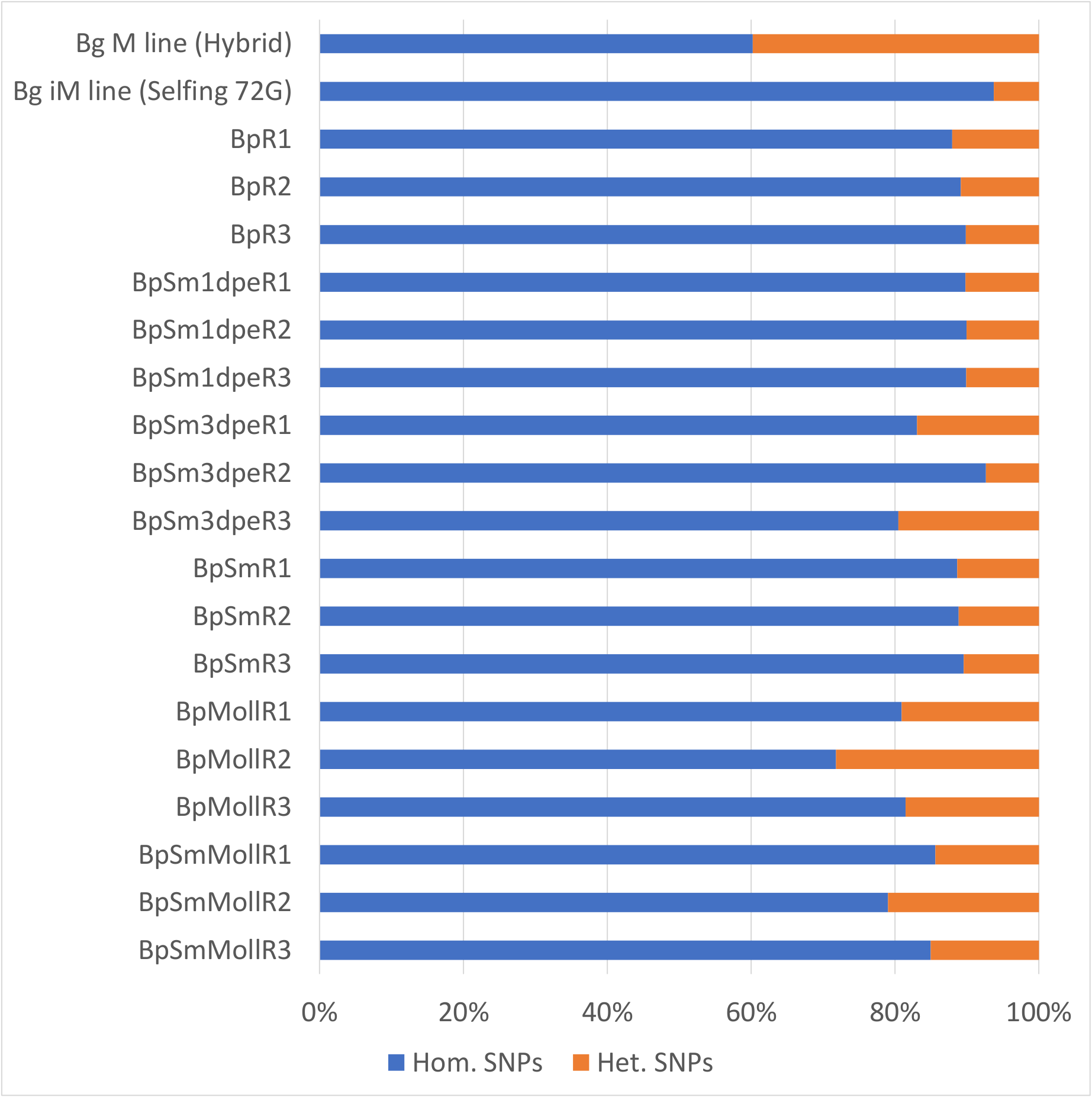

Supplement: S1 Fig — The bar shows the proportion of homozygous (blue), or heterozygous (orange) SNPs found in the individual snails used in RNA-Seq studies of B. glabrata [97] and B. pfeifferi [27,28,48]. Abbreviations: Bg, B. glabrata; Bp, B. pfeifferi; Sm, S. mansoni; dpe, days post-exposure; Moll, molluscicide treated; R#, replicate number. BpSmMollR3 means B. pfeifferi infected with S. mansoni and treated with molluscicide, the 3rd replicate. (TIF) [file pntd.0011208.s017.tif]

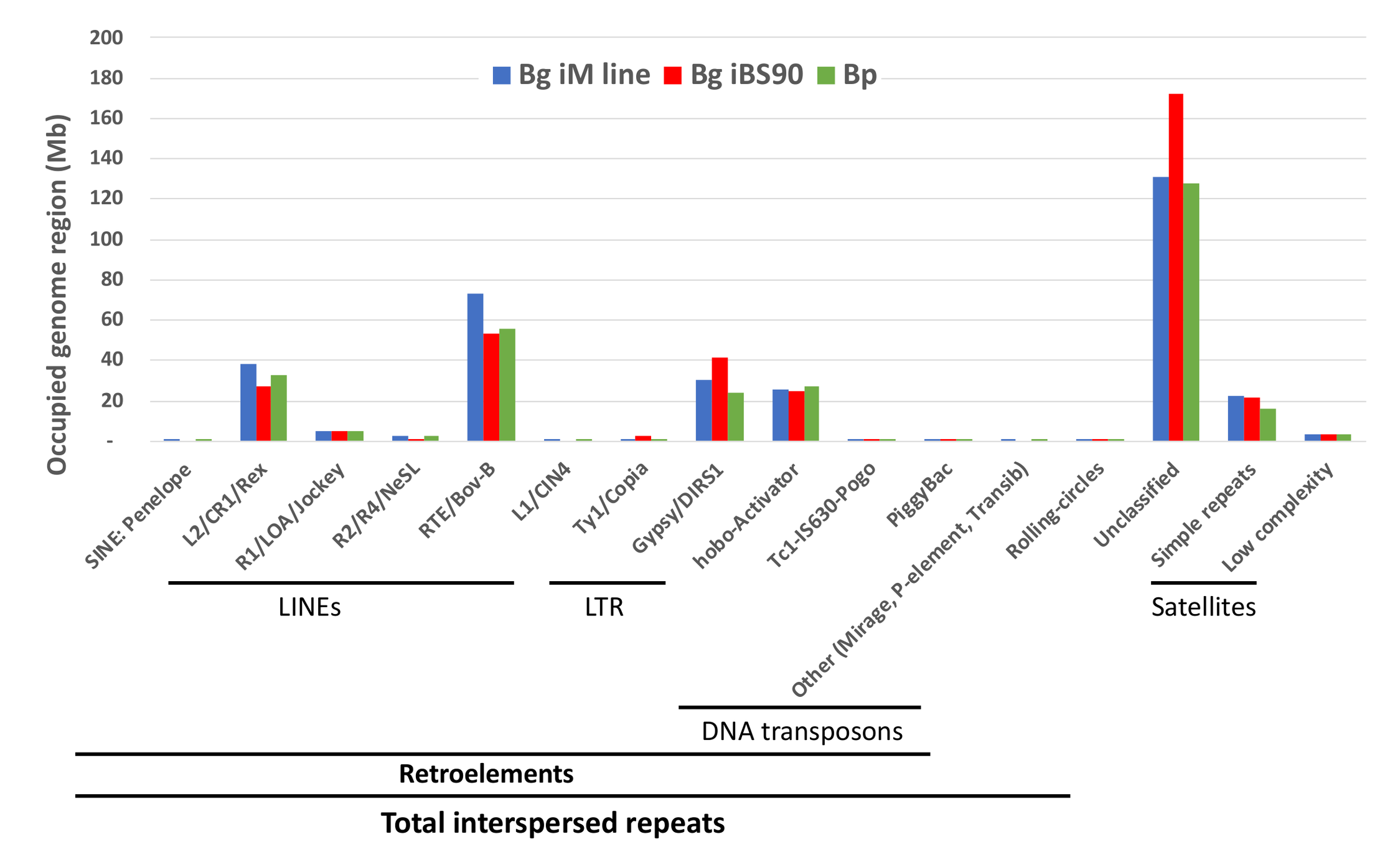

Supplement: S2 Fig — Abbreviations: Bg, B. glabrata; Bp, B. pfeifferi (TIF) [file pntd.0011208.s018.tif]

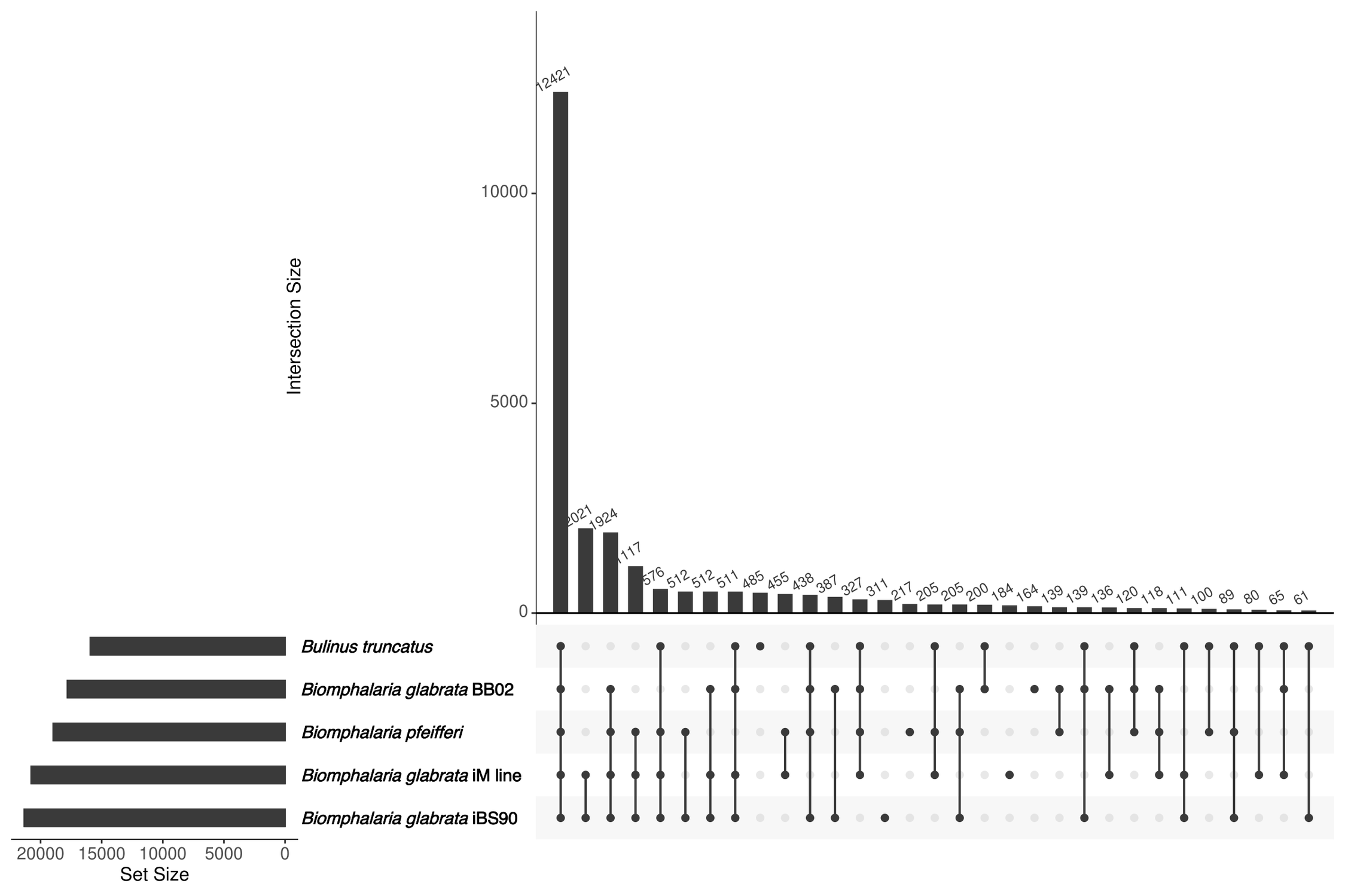

Supplement: S3 Fig — (TIF) [file pntd.0011208.s019.tif]

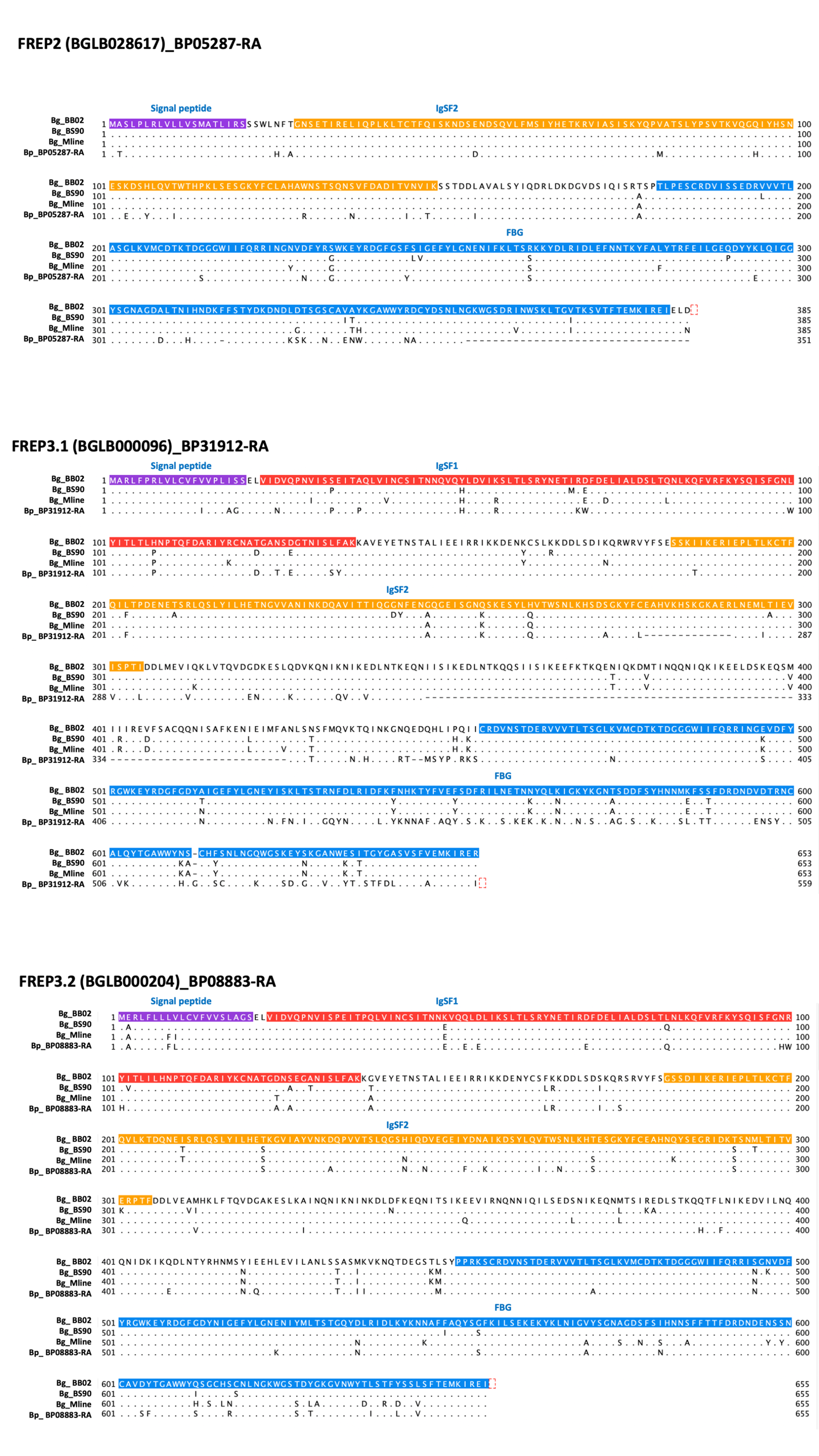

Supplement: S4 Fig — (TIF) [file pntd.0011208.s020.tif]

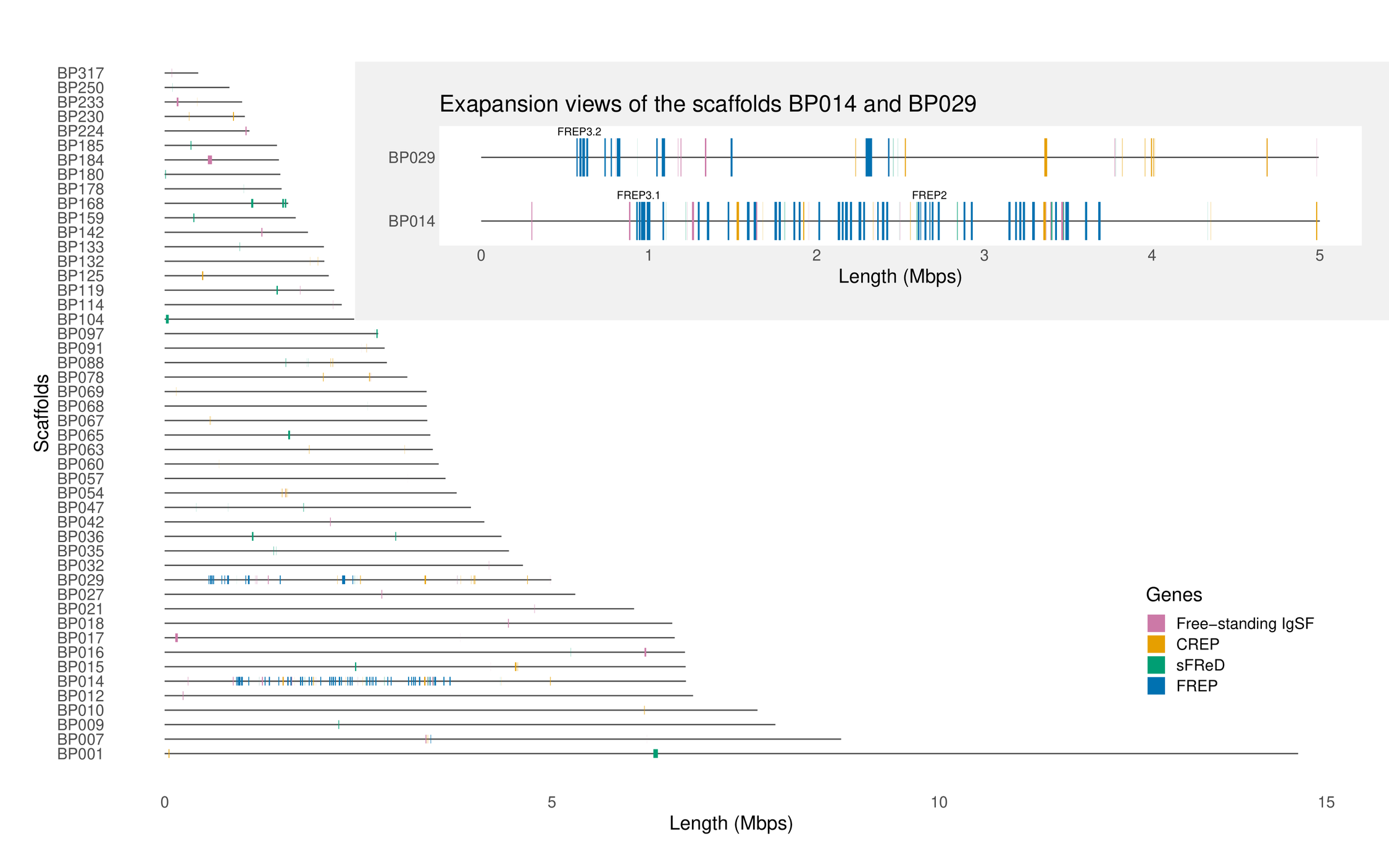

Supplement: S5 Fig — The free-standing IgSF domain-containing genes (free-standing IgSF in purple) and variable immunoglobulin and lectin domain-containing molecules (VIgLs) gene families including FREPs (blue), CREPs (yellow), and sFReDs (green) were marked on scaffolds according to predicted genomic locations. (TIF) [file pntd.0011208.s021.tif]

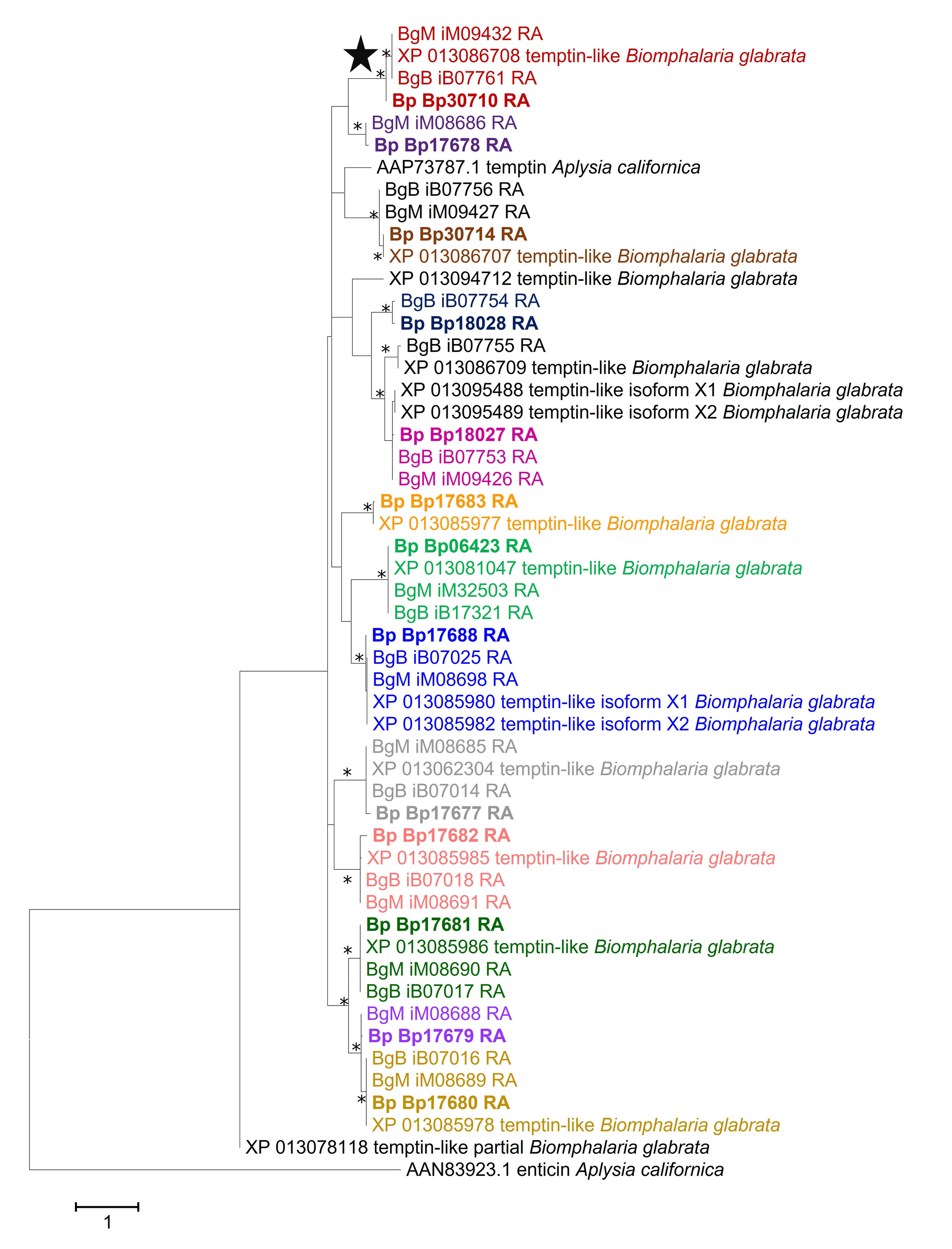

Supplement: S6 Fig — The evolutionary history was inferred by using the Maximum Likelihood method and JTT matrix-based model from Jones et al. (1992) [151]. An (*) indicates bootstrap values greater than 90%. All 13 B. pfeifferi temptin-like (including those without signal peptides) genes are shown in bold and each clade is color coded. All the B. pfeifferi temptin-like genes group with B. glabrata temptin-like genes, either from B. glabrata BB02, iM line, or iBS90 genomes. One temptin-like gene grouped with BB02, two grouped with iM line, one grouped with iBS90, and the rest of the 8 temptin-like genes all grouped together. The clade with the star is the BgTempin clade from Pila et al. (2017) [144], a study which demonstrated B. glabrata is attracted to this protein. The tree with the highest log likelihood (-1627.03) is shown. A discrete Gamma distribution was used to model evolutionary rate differences among sites (5 categories (+G, parameter = 2.4126)). The rate variation model allowed for some sites to be evolutionarily invariable ([+I], 1.09% sites). The tree is drawn to scale, with branch lengths measured in the number of substitutions per site. This analysis involved 52 amino acid sequences. All positions containing gaps and missing data were eliminated (complete deletion option). There was a total of 46 positions in the final dataset. Evolutionary analyses were conducted in MEGA X by Kumar et al., (2018) [71]. (TIF) [file pntd.0011208.s022.tif]
